# Musk Kinase Activity is Modulated By A Serine Phosphorylation Site in The Kinase Loop

**DOI:** 10.1038/srep33583

**Published:** 2016-09-26

**Authors:** B. Z. Camurdanoglu, C. Hrovat, G. Dürnberger, M. Madalinski, K. Mechtler, R. Herbst

**Affiliations:** 1 Center for Brain Research, Medical University of Vienna, Spitalgasse 4, 1090 Vienna, Austria; 2 Institute for Molecular Pathology (IMP), Dr. Bohr-Gasse 7, 1030 Vienna, Austria; 3 Institute of Molecular Biotechnology (IMBA), Dr. Bohr-Gasse 3, 1030 Vienna, Austria; 4 Gregor Mendel Institute (GMI), Austrian Academy of Sciences, Vienna Biocenter (VBC), Dr. Bohr-Gasse 3, 1030 Vienna, Austria; 5 Institute of Immunology, Medical University of Vienna, Lazarettgasse 19, 1090 Vienna, Austria

## Abstract

The neuromuscular junction (NMJ) forms when a motor neuron contacts a muscle fibre. A reciprocal exchange of signals initiates a cascade of signalling events that result in pre- and postsynaptic differentiation. At the centre of these signalling events stands muscle specific kinase (MuSK). MuSK activation, kinase activity and subsequent downstream signalling are crucial for NMJ formation as well as maintenance. Therefore MuSK kinase activity is tightly regulated to ensure proper NMJ development. We have identified a novel serine phosphorylation site at position 751 in MuSK that is increasingly phosphorylated upon agrin stimulation. S751 is also phosphorylated in muscle tissue and its phosphorylation depends on MuSK kinase activity. A phosphomimetic mutant of S751 increases MuSK kinase activity in response to non-saturating agrin concentrations . In addition, basal MuSK and AChR phosphorylation as well as AChR cluster size are increased. We believe that the phosphorylation of S751 provides a novel mechanism to relief the autoinhibition of the MuSK activation loop. Such a lower autoinhibition could foster or stabilize MuSK kinase activation, especially during stages when no or low level of agrin are present. Phosphorylation of S751 might therefore represent a novel mechanism to modulate MuSK kinase activity during prepatterning or NMJ maintenance.

The vertebrate neuromuscular junction (NMJ) represents a special chemical synapse between a motor neuron and a skeletal muscle fibre. As such the NMJ converts nerve-elicit signals into muscle contraction. The formation of the NMJ is crucially linked to signalling events induced by the receptor tyrosine kinase MuSK^1^. MuSK is activated by the motor neuron-derived heparansulfate proteoglycan agrin^2^,^3^. Agrin does not bind MuSK directly but interacts with Lrp4, a member of the LDL receptor family^4^,^5^. Agrin binding results in the formation of a tetrameric agrin-Lrp4 complex that is capable of inducing MuSK dimerization and subsequent autophosphorylation of MuSK^6^. The resulting activation of the MuSK kinase induces a signalling cascade leading to the formation of the NMJ including postsynaptic differentiation, characterized by the clustering of acetylcholine receptors (AChRs) at synaptic sites, and presynaptic differentiation as depicted by nerve terminal polarization and the development of active zones^7^. *Agrin*, *MuSK* and *lrp4* mutant mice fail to form NMJs and consequently die at birth due to respiratory failure^8^,^9^,^10^.

MuSK kinase activity is tightly regulated by the juxtamembrane Y553 and by the binding of Dok7^1^. Trans-autophosphorylation of Y553 potentiated by Lrp4 recruits Dok7 to the juxtamembrane NPXY motif. Dok7 dimerizes via its PH domain and thus juxtaposes two MuSK kinase domains for further trans-phosphorylation and autoactivation. Several lines of evidence have shown that MuSK kinase activity and MuSK scaffolding ability are crucial for NMJ formation: (1) expression of MuSK mutants with a defective kinase domain inhibits agrin-induced AChR clustering^11^; (2) tyrosine kinase inhibitors block agrin-induced AChR clustering^12^; (3) specific residues in the MuSK cytoplasmic domain, in particular a NPXY motif in the juxtamembrane region, are required for down-stream signalling^13^,^14^; (4) several molecules including scaffolding proteins, adaptor proteins, kinases and phosphatases have been identified that are downstream from MuSK^1^. These results together with the above-mentioned data put MuSK at the centre of signal transduction events that result in the formation of mature and functional NMJs.

To better understand the signalling network initiated by MuSK we have recently performed a quantitative phosphoproteomics screen to identify targets downstream of MuSK^15^. Within upregulated phosphopeptides we identified peptides carrying a phosphorylated serine at position 751. S751 is located in the activation loop of the MuSK kinase domain, in close proximity to the critical tyrosine residues 750, 754 and 755. Interestingly, agrin-induced S751 phosphorylation lagged agrin-induced phosphorylation of the juxtamembrane Y553. S751 phosphorylation was also observed in muscle tissue. Mutation of S751 to mimic phosphorylation or to eliminate phosphorylation did not affect MuSK tyrosine phosphorylation in response to saturating agrin levels. However, a phosphomimetic mutant increased basal MuSK phosphorylation and phoshorylation upon stimulation with non-saturating levels of agrin. Consequently basal AChR phosphorylation was increased as well as the temporal response to agrin treatment. In addition, we found an increased AChR cluster size in response to agrin further supporting the notion that S751 modulates MuSK kinase activity upon agrin stimulation.

## Results

### S751 phosphorylation in response to agrin

**Figure 1:**
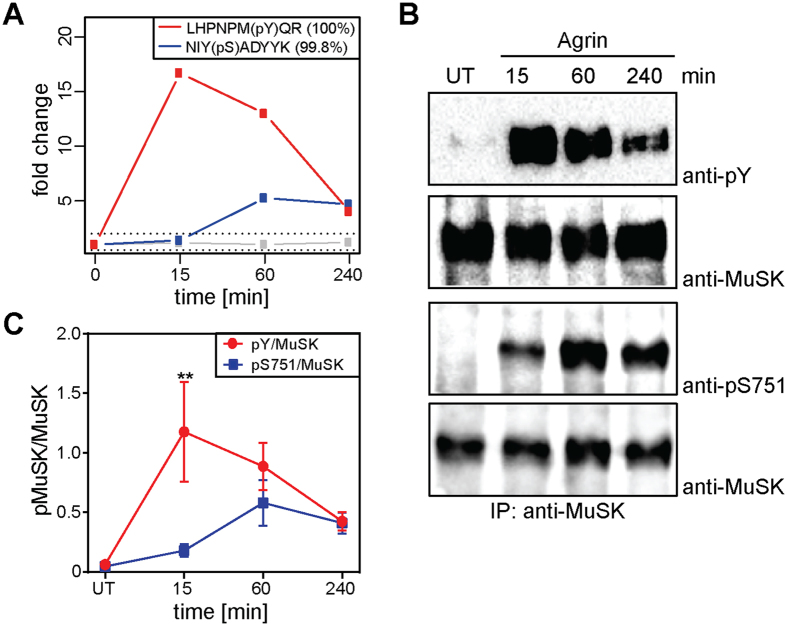
S751 is phosphorylated in response to agrin. (**A**) Phosphopeptides were identified by quantitative MS analysis. Quantification of these showed induction of pY553 (red) and pS751 (blue) upon agrin treatment. In addition, phosphorylation of MuSK S678 was detected but not significantly regulated (grey). Phosphosites of regulated peptides were localized with high confidence (phosphoRS site probabilities are indicated in brackets). (**B**) Cell lysates from agrin-stimulated muscle cells were subjected to immunoprecipitation with anti-MuSK antibodies. Samples were analysed by immunoblotting using antibodies against phospho-tyrosine, phospho-S751 and MuSK. (**C**) Quantification of immunoblots shows phosphorylation kinetics similar to the kinetics of MS analysis. Values are presented as the mean ± S.E.M. (two-way ANOVA with Sidak’s multiple comparison tests, n = 5, p = 0.004). Full-length blots are presented in Supplementary Fig. S7. IP, immunoprecipitation; pY, phospho-tyrosine; UT, untreated.

In a previous study we have used a muscle C2C12 cell culture model system to identify and characterize the phosphoproteome during agrin-induced MuSK activation. We were able to show that MuSK induces the phosphorylation of a large set of proteins with a distinct temporal manner of regulation^15^. In total we identified 152 proteins whose phosphorylation was either up- or downregulated at least two-fold. As expected, MuSK itself was among these regulated phosphoproteins. Phosphopeptides carrying Y553, the major phosphorylation site in MuSK, were detected in this quantitative mass spectrometry (MS) analysis (Fig. 1A). Y553 was rapidly and transiently phosphorylated. Phosphorylation peaked at 15 minutes (17-fold induction) and was reduced close to basal level at 240 minutes. In addition to MuSK tyrosine phosphorylation, we identified a novel phosphorylation site on S751, which is upregulated during late agrin stimulation (Fig. 1A). The peptide carrying S751 showed high isolation interference but identification and phosphorylation site localization was achieved with high confidence (Supplementary Fig. S1). Since the majority of phosphopeptides (98%) in our analysis were unregulated, we argue that the interfering peptides were also unregulated. Under this assumption S751 phoshorylation peaked at 60 minutes (five-fold induction) and remained stably phosphorylated until 240 minutes. To confirm this observation we stimulated C2C12 myotubes with agrin for different time periods, isolated MuSK via immunoprecipitation from cell lysates and performed immunoblotting with antibodies directed against phosphorylated S751 and phospho-tyrosine, respectively (Fig. 1B). Quantification revealed phosphorylation kinetics similar to the kinetics identified in the MS analysis (Fig. 1C). S751 is located in the activation loop of the MuSK kinase domain between Y750 and Y754/Y755, which are critical for MuSK activation. The specific kinetics of S751 phosphorylation and its location suggest a role during MuSK activation and/or signalling.

### MuSK phosphorylated at S751 is present at NMJs

**Figure 2:**
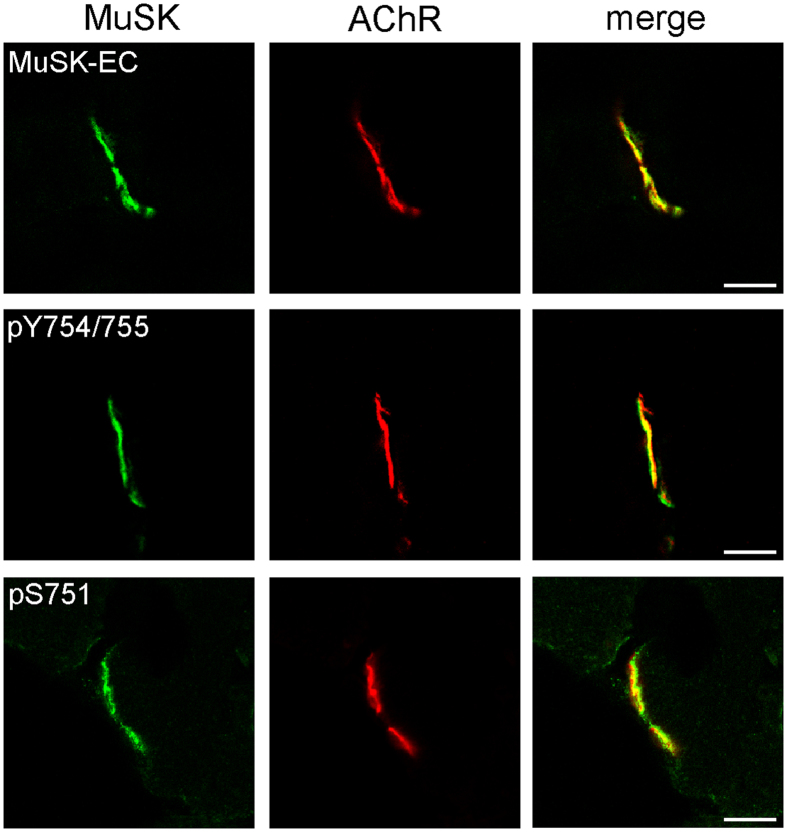
Phosphorylated S751 is present at NMJs. Frozen sections of *M. gastrocnemius* were stained with MuSK specific antibodies (green) and with Alexa 594-conjugated α-BGT (red) to label AChRs. MuSK-EC recognizes an epitope of the MuSK extracellular domain, pY754/755 recognizes an epitope carrying phosphorylated Y754 and Y755 and pS751 is directed against an epitope containing phosphorylated S751. Note that S751 phosphorylation co-localizes with AChRs. Images were obtained by confocal microscopy and representative images are shown. Scale bar, 10 μm. EC, extracellular.

Next we asked whether S751 is also phosphorylated in MuSK *in vivo*. We stained muscle sections with antibodies against pS751. Parallel sections were stained with antibodies against MuSK or antibodies against pY754/Y755 of the MuSK activation loop. All samples were co-stained with α-BGT to label AChRs. Figure 2 shows that antibodies against pS751 specifically marked the postsynaptic region, which was identified as AChR-positive region. Antibodies against MuSK or pY754/Y755 presented a similar staining pattern as anti-pS751 antibodies. Pre-incubation of anti-pS751 antibodies with the peptide representing the pS751 epitope abolished the staining of NMJs, whereas pre-incubation with non-phoshorylated epitope did not interfere with antibody binding (Supplementary Fig. S2). In addition, localization and signal of pS751 was similar in different muscles (sternomastoid, gastrocnemius and intercostal, data not shown). We therefore conclude that S751 is phosphorylated *in vivo* and that MuSK carrying pS751 is present at NMJs.

### S751 modulates basal MuSK tyrosine phosphorylation and subsequent AChR phosphorylation

**Figure 3:**
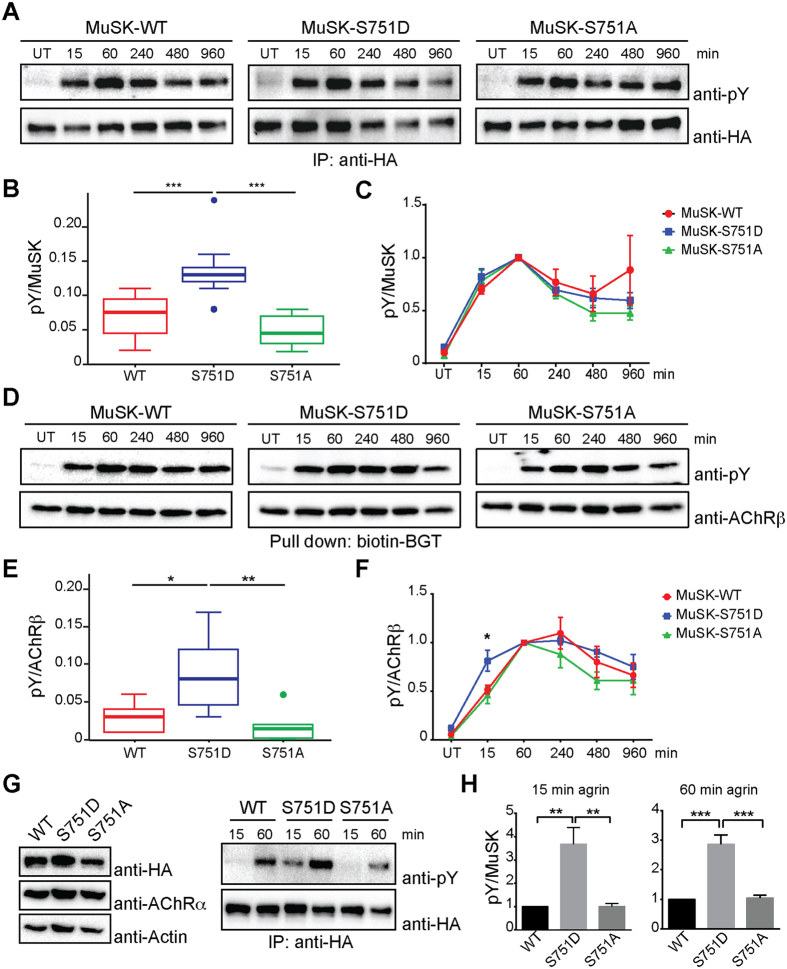
A phosphomimetic S751 mutation modulates MuSK phosphorylation and causes increased AChR phosphorylation. Myotubes expressing MuSK wild-type or S751 mutants were stimulated with agrin for different time periods and cell lysates were processed for further analysis. (**A**) MuSK was immunoprecipitated with anti-HA antibodies and assayed by immunoblotting using antibodies against phospho-tyrosine and HA, respectively. (**B**) Quantification of MuSK phosphorylation in the absence of agrin is shown. Mutation S751D increases basal MuSK phosphorylation compared to S751A (p = 0.00001) and WT (p = 0.00079). Values are presented as the median ± interquartile range (IQR). Outliers are plotted as individual points (n = 9). (**C**) The kinetics of MuSK phosphorylation in response to agrin is shown. MuSK phosphorylation after 1 h agrin stimulation is set to 1 and time points are quantified accordingly for each data set. Values are presented as the mean ± S.E.M. (n = 9). (**D**) AChRs were affinity-purified using biotin-α-BGT and assayed by immunoblotting using anti-phospho-tyrosine and anti-AChR β antibodies, respectively. (**E**) AChR β; phosphorylation in the absence of agrin was quantified. Phosphorylation increases with MuSK-S751D compared to MuSK-S751A (p = 0.0092) and MuSK-WT (p = 0.0200). Values are presented as the median ± IQR. Outliers are plotted as individual points (n = 6). (**F**) The kinetics of AChR β; phosphorylation upon agrin stimulation was quantified as in (C). Phosphorylation in cells expressing MuSK-S751D is increased after 15 min agrin stimulation compared to MuSK-S751A (p = 0.0228) and MuSK-WT (p = 0.0498). Values are presented as the mean ± S.E.M. (n = 6). (**G**) Cells were stimulated with non-saturating concentrations of agrin and processed as in (A). Total lysates were analysed with antibodies against HA, AChR α and Actin, respectively. (**H**) Quantification of MuSK phosphorylation is shown (MuSK-WT set to 1). Mutation S751D increases MuSK phosphorylation compared to S751A (15 min: p = 0.0036; 60 min: p = 0.005) and WT (15 min: p = 0.0035; 60 min: p = 0.004); n = 4. One-way ANOVA with Tukey’s multiple comparison test was used. Full-length blots are presented in Supplementary Fig. S8. IP, immunoprecipitation; pY, phospho-tyrosine; UT, untreated; WT, wild-type.

Muscle cells lacking MuSK are unable to respond to agrin and fail to form AChR clusters^2^,^11^. We used MuSK^−/−^ muscle cells to study S751 during MuSK activation and downstream signalling^2^,^13^. We mutated the serine (S) to alanine (A) to generate a phosphorylation-deficient mutant and to aspartate (D), which can mimic phosphorylation to generate a phosphorylation-active mutant. *MuSK*^*−/−*^ muscle cell lines expressing these S751 mutants or MuSK wild-type were generated, differentiated into myotubes and stimulated with agrin (Fig. 3). To examine MuSK activation we isolated MuSK and to study MuSK downstream signalling we isolated AChRs from cell lysates. Phosphorylation and expression were analysed by immunoblotting. Cell lines expressed similar levels of MuSK and AChR (Supplementary Fig. S3). As shown in Fig. 3B, a phosphomimetic mutant, generated by a S751D mutation, increased the basal tyrosine phosphorylation level of MuSK in the absence of agrin compared to MuSK S751A and MuSK wild-type. The temporal activation profile of MuSK tyrosine phosphorylation in response to agrin was similar for MuSK wild-type, MuSK S751D and MuSK S751A (Fig. 3C). Consistent with these findings the basal phosphorylation of AChR β; was increased in muscle cells expressing MuSK S751D (Fig. 3D,E). Moreover, AChR β; phosphorylation after short agrin stimulation (15 minutes) was significantly increased in muscle cells expressing MuSK S751D compared to cells expressing MuSK S751A or MuSK wild-type (Fig. 3F).

**Figure 4:**
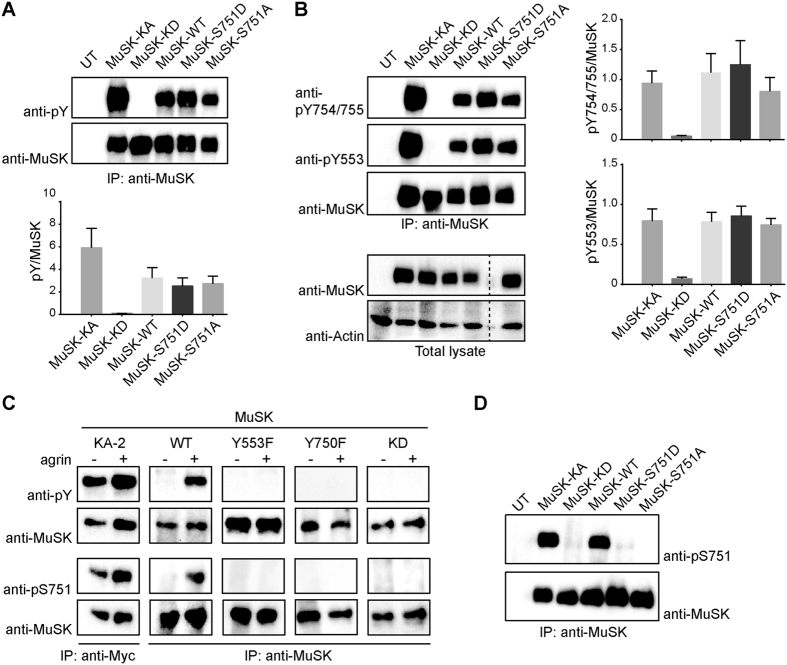
S751 does not regulate autoactivation of MuSK in heterologous cells and phosphorylation of S751 is dependent on MuSK kinase activity. (**A**) HEK 293T cells were transiently transfected with wild-type and mutant MuSK constructs resulting in autoactivation of MuSK kinase. Cell lysates were subjected to immunoprecipitation (IP) using anti-MuSK antibodies and immunoblotted with anti-phospho-tyrosine (pY) and anti-MuSK antibodies, respectively. Wild-type MuSK (WT) is strongly tyrosine phosphorylated. Kinase-dead MuSK (KD) lacks tyrosine phosphorylation whereas phosphorylation of kinase-active MuSK (KA) is further increased compared to wild-type. S751 mutants are similarly autoactivated as MuSK wild-type. Values are presented as the mean ± S.E.M. (Kruskal-Wallis with Dunn’s multiple comparison test, n = 5). (**B**) HEK 293T cells were transiently transfected with wild-type and mutant MuSK constructs resulting in autoactivation of MuSK kinase. Cell lysates were subjected to immunoprecipitation (IP) using anti-MuSK antibodies and immunoblotted using antibodies against MuSK pY553, MuSK pY754/755 and MuSK, respectively. Total lysates were analysed with antibodies against MuSK and Actin, respectively. Phosphorylation of juxtamembrane Y553 and Y754, Y755 of the kinase loop occur at similar level in S751 mutant MuSK compared to MuSK-wild-type. Values are presented as the mean ± S.E.M. (Kruskal-Wallis with Dunn’s multiple comparison test, n = 5). (**C**) Muscle cells expressing MuSK kinase mutants were stimulated with agrin (+, A4B8; −, A0B0) and cell lysates were subjected to immunoprecipitation (IP) using anti-MuSK or anti-Myc antibodies. Samples were analysed by immunoblotting using antibodies against phospho-tyrosine (PY), MuSK pS751 and MuSK, respectively. Kinase-dead MuSK (KD), MuSK Y750, 754, 755F (Y750F) and MuSK Y553F are unresponsive to agrin. Wild-type MuSK (WT) is phosphorylated in response to agrin and kinase-active MuSK (KA-2) is highly phosphorylated in the presence and absence of agrin. Note that phosphorylation of S751 depends on MuSK tyrosine phosphorylation. (**D**) HEK 293T were transiently transfected with wild-type and mutant MuSK constructs resulting in autoactivation of MuSK kinase. Cell lysates were subjected to immunoprecipitation (IP) using anti-MuSK antibodies and immunoblotted with anti-MuSK pS751 and anti-MuSK antibodies, respectively. Phosphorylation of S751 is only detected upon autoactivation of the MuSK kinase. Full-length blots are presented in Supplementary Fig. S9.

Next, we investigated the autoactivation of MuSK in heterologous cells by expressing wild-type and mutant MuSK in HEK 293T cells. Overexpression of MuSK induces dimerization of MuSK and its autophosphorylation. Figure 4A shows that MuSK wild-type was robustly tyrosine phosphorylated. Autophosphorylation was abolished in mutant MuSK protein that contained a K608A substitution in the autocatalytic loop^16^,^11^,^14^. In contrast, autophosphorylation was further increased in a kinase-active mutant that carried the mutations LS746/747MT^17^. Autophoshorylation was not significantly altered in MuSK S751A and MuSK S751D compared to wild-type MuSK. Similarly, when we used phospho-specific antibodies against pY553 and pY754/755 we observed no significant change in phosphorylation in MuSK S751A and MuSK S751D compared to wild-type MuSK (Fig. 4B). Control experiments demonstrated that the expression level of the different constructs was similar (Supplementary Fig. S4). These results suggest that S751 modulates basal agrin-independent MuSK phosphorylation in muscle cells and subsequently promotes early AChR phosphorylation without affecting MuSK autoactivation.

### A phosphomimetic mutant of S751 increases MuSK phosphorylation in response to sub-threshold levels of agrin

Increased basal phosphorylation of MuSK S751D might be caused by a lowered autoinhibition of MuSK, thereby increasing trans-phosphorylation and kinase activity. Phosphorylation of S751, and as such a S751 phosphomimetic, might relief autoinhibition to foster MuSK kinase activation in the absence of agrin or when low levels of agrin are present. To test this, we stimulated myotubes expressing MuSK wild-type, MuSK S751D or MuSK S751A with non-saturating concentrations of agrin for 15 and 60 minutes. Cells were extracted, subjected to immunoprecipitation and analysed by immunoblotting. Figure 3G,H show that MuSK S751D presents a significantly increased phosphorylation compared to MuSK wild-type and MuSK S751A. We conclude that phoshorylation of S751 represents a positive modulatory event that enhances MuSK phoshorylation and activity in response to non-saturating concentrations of agrin, which might represent a means to overcome strong autoinhibition.

### S751 phosphorylation depends on MuSK kinase activity

We used muscle cells expressing MuSK carrying mutations that affect MuSK kinase activity to study S751 phosphorylation upon agrin stimulation. MuSK Y750, 754, 755F (termed Y750F) and MuSK kinase-dead (KD) lack kinase activity due to mutations that interfere with activation of the kinase^11^,^13^,^14^. MuSK Y553F is not activated since mutation of the juxtamembrane Y553 strengthens the inhibition of the kinase activity via the juxtamembrane region^13^,^18^. In addition, Dok7 cannot bind, which is crucial for full activation of MuSK^19^,^20^. Kinase-active MuSK KA-2 has the MuSK transmembrane region replaced by the Neu transmembrane region (NeuT)^21^. A mutation in the NeuT region leads to a constitutive dimerization and consequent activation of the receptor. As previously shown^13^,^14^, MuSK KD, MuSK Y750F and MuSK Y553F were not activated by agrin and lack tyrosine phosphorylation (Fig. 4C). In contrast, MuSK wild-type was efficiently phosphorylated in response to agrin and MuSK KA-2 was strongly phosphorylated independent of agrin. Phosphorylation of S751 correlated with MuSK tyrosine phosphorylation (Fig. 4C). pS751 was only observed in cells expressing MuSK wild-type or MuSK KA-2 demonstrating that MuSK kinase activity is required for S751 phosphorylation. Interestingly, we observed the same interconnection between MuSK tyrosine phosphorylation and S751 phosphorylation in HEK 293T cells (Fig. 4A,D). These data provide evidence that S751 phosphorylation requires MuSK kinase activity and that this phosphorylation is neither agrin-dependent nor muscle-specific.

### Surface expression and stability of MuSK carrying mutations in S751

**Figure 5:**
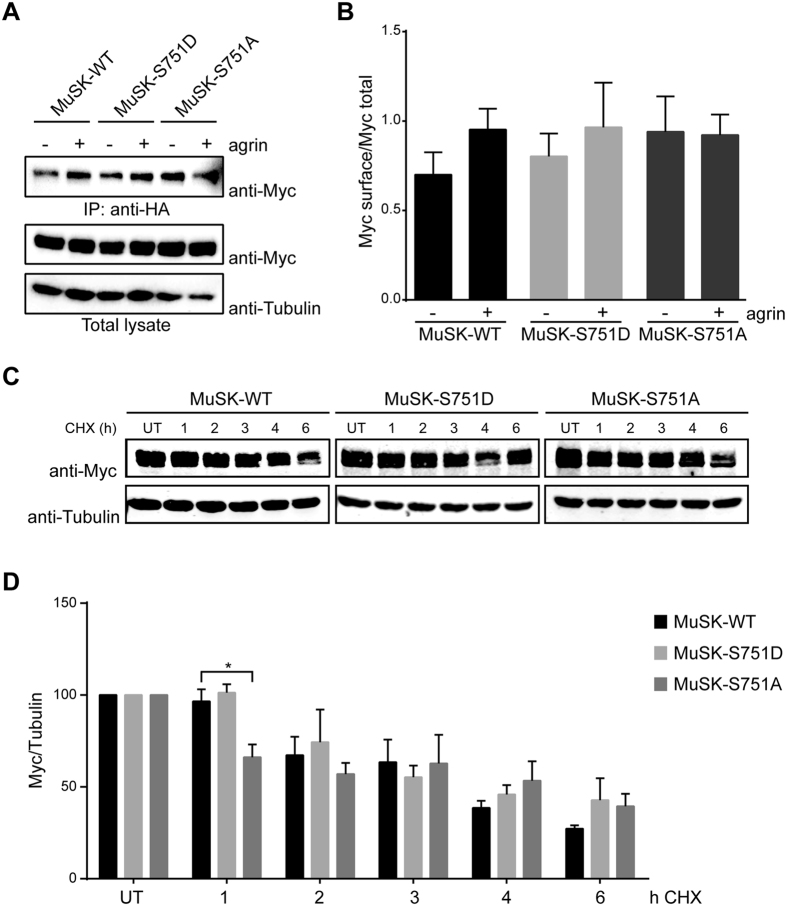
Mutation of S751 does not alter MuSK surface expression and MuSK protein stability. (**A**) MuSK wild-type (WT), S751D, S751A constructs with a N-terminal HA-Tag and a C-terminal Myc-Tag were expressed in C2C12 muscle cells. Differentiated myotubes were stimulated with agrin for 30 min (+, A4B8; −, A0B0) and incubated with anti-HA antibodies to label surface MuSK. Cells were lysed and HA-bound MuSK protein was purified by immunoprecipitation (IP). Samples were analysed by immunoblotting using anti-Myc antibodies. Total lysates were used as loading controls and immunoblotted with antibodies against Myc and Tubulin, respectively. (**B**) Quantification of MuSK surface expression normalized to total MuSK protein is shown. Cell lines express similar levels of surface MuSK. MuSK wild-type and MuSK S751D show slightly increased MuSK surface expression upon agrin treatment compared to MuSK S751A. Values are presented as the mean ± S.E.M. (one-way ANOVA with Tukey’s multiple comparison test, n = 4) (**C**) C2C12 myotubes expressing MuSK wild-type (WT), S751D or S751A with a N-terminal HA-Tag and a C-terminal Myc-Tag were incubated with cycloheximide (CHX) for different time periods. Cells were lysed and total lysates were analysed by immunoblotting using antibodies against anti-Myc and anti-Tubulin antibodies, respectively. Note that MuSK expression is highly decreased after 6 h of cycloheximide incubation whereas Tubulin is stable. (**D**) Quantification of MuSK expression upon cycloheximide treatment is shown (normalized against Tubulin). MuSK-S751A is significantly faster degraded within the first hour of cycloheximide treatment compared to MuSK–WT (p = 0.044) and MuSK-S751D (p = 0.017). In contrast, expression level is similar for all tested MuSK proteins at later time points. Values are presented as the mean ± S.E.M. (two-way ANOVA with Tukey’s multiple comparison test, n = 3 in duplicates). Full-length blots are presented in Supplementary Fig. S10.

Serine phosphorylation of RTKs not only regulates downstream signalling but also endocytosis and internalization of RTKs^22^,^23^,^24^,^25^. To examine whether S751 phosphorylation influences MuSK surface expression we expressed MuSK wild-type, MuSK S751A and MuSK S751D with a N-terminal HA-Tag in C2C12 muscle cells. Surface MuSK was isolated from myotubes untreated or treated with agrin via the HA-Tag. Subsequent analysis of protein expression by immunoblotting revealed that similar amounts of MuSK wild-type, MuSK S751A and MuSK S751D were present on the cell surface (Fig. 5A,B). This observation is further supported by determining MuSK surface expression using biotinylation of surface proteins (Supplementary Fig. S5). Establishing that mutations in S751 did not affect surface expression, we next tested whether MuSK dimerization is affected. Here we took advantage of the fact that our cell system expressed endogenous wild-type MuSK and exogenous MuSK either wild-type, S751A or S751D, which carry, in addition to the N-terminal HA-Tag, a C-terminal Myc-Tag. We used an antibody that recognizes only endogenous MuSK for immunoprecipitation. Samples were subsequently analysed by immunoblotting against endogenous MuSK using anti-MuSK antibodies and exogenous MuSK using antibodies against the Myc-Tag. Quantification showed that similar amounts of wild-type and mutant MuSK were isolated together with endogenous MuSK suggesting that MuSK dimerization is not affected by S751 (Supplementary Fig. S6).

In a different set of experiments we asked whether mutation of S751 has an effect on MuSK protein stability. For this, we treated myotubes with cycloheximide to inhibit protein synthesis followed by an analysis of protein expression by immunoblotting. As shown in Fig. 5C, MuSK protein expression started to decrease already after 2 hours of cycloheximide incubation and was strongly decreased by 6 hours of cycloheximide treatment. In comparison, tubulin was stable during this time period. Quantification showed that MuSK wild-type has a half-life of about 4 hours (Fig. 5D). This was also true for MuSK S751A and MuSK S751D. Surprisingly, MuSK S751A was significantly faster degraded within the first hour of cycloheximide treatment but showed a similar expression level at later time points. Taken together we conclude that S751 is not involved in MuSK protein folding or stability since neither MuSK surface expression, nor dimerization or protein stability is affected.

### Expression of MuSK S751D increases AChR cluster size

**Figure 6:**
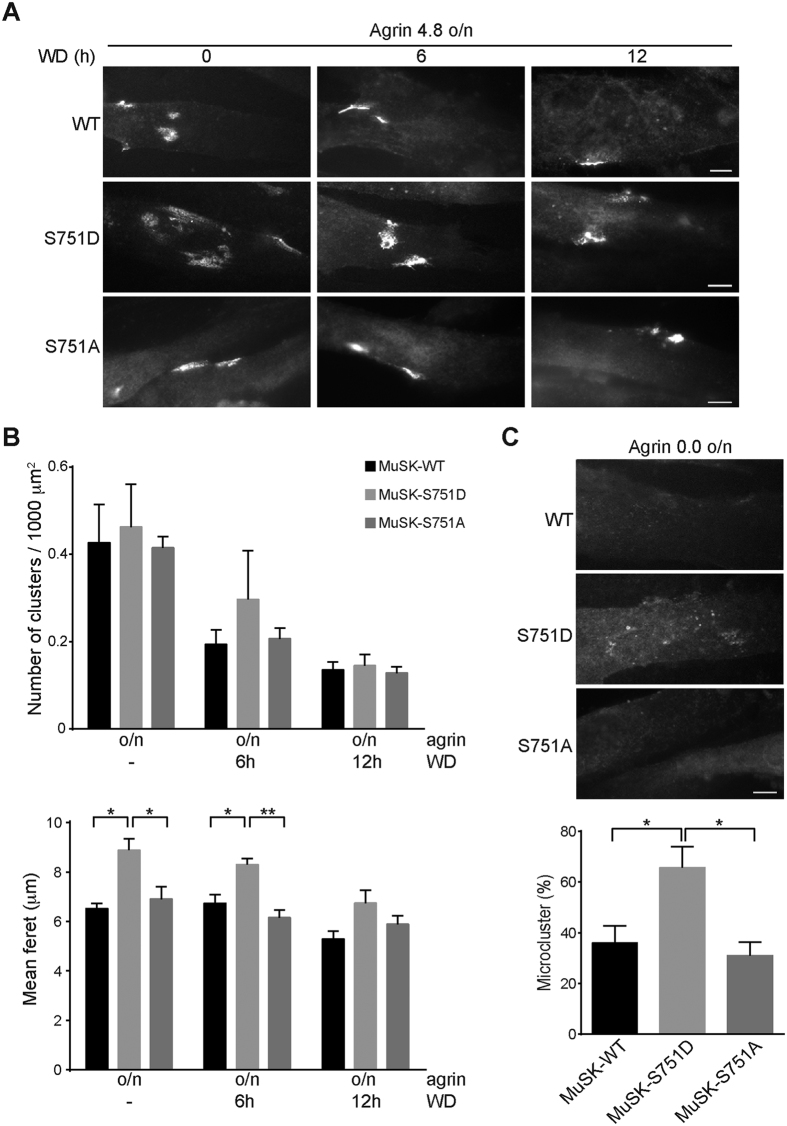
A MuSK S751 phosphomimetic mutant increases the size of AChR clusters but does not increase the number of AChRs. (**A**) *MuSK*^−/−^ muscle cells expressing MuSK wild-type and S751 mutant proteins were differentiated and stimulated with agrin for 16 h (overnight) to induce AChR cluster formation. To measure AChR cluster stability, agrin was removed and the cells were maintained in agrin-free medium for indicated times. Representative images of clusters stained with Alexa 594-conjugated α-BGT are shown. Scale bar, 10 μm. (**B**) The number of AChR clusters normalized to myotube area (Kruskal-Wallis with Dunn’s multiple comparison test, mean ± S.E.M., n = 4 with > 75 myotubes in total for each treatment) and the mean feret of clusters (one-way ANOVA with Tukey’s multiple comparison test, mean ± S.E.M., *p < 0.05, **p < 0.01, n ≥ 100 from four different experiments) were quantified using ImageJ. Note that AChR clusters are significantly bigger in cells expressing MuSK-S751D after agrin stimulation and after 6 h of agrin withdrawal. (**C**) Graph showing the percentage of microclusters per myotube. MuSK-S751D expressing cells form more microclusters compared to cells expressing MuSK-S751 A (p = 0.0270) and MuSK-WT (p = 0.0382). Values are presented as the mean ± S.E.M. (one-way ANOVA with Tukey’s multiple comparison test, n = 3 with > 100 myotubes in total for each cell line). WD, withdrawal; o/n, overnight; WT, wild-type.

The hallmark of MuSK signalling is the clustering of AChRs to discrete patches on the muscle membrane. Changes in MuSK kinase activity or alterations in the downstream signalling pathway greatly influence AChR clustering^11^,^13^,^14^,^16^. Defects in AChR clustering usually result in increased or decreased number of clusters or increased or decreased size of clusters. We stimulated myotubes expressing MuSK wild-type, S751A or S751D with neural agrin A4B8 overnight followed by staining of AChRs with α-BGT. Figure 6A shows that AChR clusters were formed in all cell lines. To examine AChR cluster stability we removed agrin and cultured the cells for an additional 6 or 12 hours in agrin-free medium. Again we stained AChR clusters with α-BGT. AChR clusters decreased in number and size upon agrin withdrawal (Fig. 6A). As control we treated myotubes overnight with muscle agrin A0B0, which does not activate MuSK and therefore fails to induce AChR clusters^13^,^21^. Number and size of AChR clusters were subsequently analysed (Fig. 6B). Mutation of S751 either to alanine or aspartate did not affect the ability of myotubes to form AChR clusters. The number of clusters was similar in all cell lines after overnight agrin stimulation as well as after agrin withdrawal. However, when we measured AChR cluster size we found that myotubes expressing MuSK S751D formed bigger clusters upon agrin treatment compared to myotubes expressing MuSK wild-type or S751A. Moreover, clusters in MuSK S751D expressing myotubes remained big after 6 hours agrin removal compared to MuSK wild-type and MuSK S751A expressing cells. In addition, more MuSK S751D expressing myotubes formed microclusters compared to myotubes expressing MuSK wild-type or MuSK S751A (Fig. 6C). These data suggest that the increased basal kinase activity of MuSK S751D positively modulates AChR clustering.

## Discussion

MuSK kinase activity plays a crucial role during AChR clustering and NMJ formation^2^,^8^,^26^. A tight regulation of kinase activity is therefore important for the orchestration of signalling events that lead to the formation of a mature and fully functional NMJ. A lack of MuSK kinase abolishes AChR clustering whereas constitutive active MuSK clusters AChRs in the absence of agrin *in vitro*^13^,^14^,^16^. *In vivo* constitutive active MuSK induces ectopic postsynaptic structures reminiscent of NMJs independent of nerve and agrin^21^. Most importantly, mutations that affect MuSK kinase activity are causally involved in congenital myasthenic syndromes^26^,^27^,^28^,^29^. Therefore, identification of mechanisms that regulate the activity of the MuSK kinase is of great importance for NMJ development.

It was previously reported that MuSK is phosphorylated on six of the nineteen intracellular tyrosine residues *in vitro* and *in vivo*: the juxtamembrane tyrosine Y553, tyrosines Y750, Y754, and Y755 within the activation loop, Y576 near the beginning of the kinase domain and Y812 within the C-terminal loop of the kinase domain^30^. Y553 in the juxtamembrane domain and the three tyrosines in the activation loop are primary sites for MuSK kinase activity^30^. Mutation of juxtamembrane Y553 impairs activation loop autophosphorylation in response to agrin as well as intracellular downstream signalling events of agrin such as AChR phosphorylation and AChR clustering^13^. Y553 resides in an NPXY sequence motif and upon phosphorylation serves as binding site for Dok7, which facilitates trans-autophosphorylation of the activation loop via MuSK dimerization and at the same time confers downstream signalling by binding to Crk/Crkl^19^,^31^. Phosphorylation of Y750, Y754 and Y755 is required to achieve full kinase activation and mutation of all three activation loop tyrosines completely abolishes AChR clustering^13^,^18^. The function of Y576 and Y812 is less well understood. Mutation of Y576 decreases AChR clustering whereas mutation of Y812 has no effect^13^. Using a quantitative phosphoproteomics approach we identified phosphopeptides carrying Y553, S751 and S678^15^. Y553 was rapidly and highly induced upon agrin stimulation with a peak at 15 minutes. The fast and robust phosphorylation conforms to previous studies and correlates with the mode of tyrosine phosphorylation observed in response to agrin. S678, which was previously identified in MuSK subjected to *in vitro* phosphorylation, was unregulated and remained stable during all time points of agrin stimulation^30^. In contrast, S751 was phosphorylated late during agrin stimulation. The peak of phosphorylation was at 60 minutes agrin treatment and was still high after 240 minutes. Our observations from the MS analysis were further supported using antibodies that specifically recognize pS751. Most importantly, we were able to detect pS751 co-localized with AChRs in different muscles. S751 phosphorylation is dependent on MuSK tyrosine phosphorylation and activation since phosphorylation of S751 is absent in muscle cells and heterologous cells that express MuSK mutants affecting kinase activity. In contrast, constitutively-active MuSK induces S751 phosphorylation in the absence of agrin.

S751 lies within the activation loop between the critical tyrosines Y750, Y754 and Y755. The sequence motif surrounding S751 does not conform to motifs of well-known serine/threonine kinases such as PKA/G/C, proline directed MAPK (ERK1/2), JNK, CK1/2 or GSK3^32^,^33^. Using kinase inhibitors to inhibit MEK1/2 (U0126), JNK (SP600125), p38 (SB203580), ERK1/2 (K252a), PI3K (LY2942002), PKA/C/G (H7) and specific PKC subtypes (Gö6850) we were not able to inhibit specifically S751 phosphorylation without affecting MuSK tyrosine phosphorylation (data not shown). Interestingly, when we inhibited tyrosine phosphates with pervanadate we observed an increase of MuSK tyrosine phosphorylation but a decrease in S751 phosphorylation. These data indicate that the kinase responsible for S751 phosphorylation is activated by dephosphorylation.

Serine phosphorylation sites with different functional roles modulating receptor kinase activation, receptor endocytosis or internalization, expression, cell survival, proliferation and differentiation have been discovered in different RTKs. It has been reported for several RTKs such as insulin receptor, platelet-derived growth factor receptor and estrogen receptor that serine phosphorylation regulates kinase activity^34^,^35^,^36^. Additionally, the serine phosphorylation of the epidermal growth factor (EGFR), hepatocyte growth factor receptor and fibroblast growth factor receptor have been implicated in receptor endocytosis^22^,^23^,^24^,^25^. To characterize the role of S751 phosphorylation during MuSK signalling we used *MuSK*^−/−^ muscle cells expressing wild-type, phosphomimetic S751D and phosphorylation-deficient S751A MuSK. Our data suggest that S751 has a modulatory role during MuSK activation and subsequent signalling. This is based on the observations that (1) Mutation of S751 to D751 increases basal MuSK phosphorylation. (2) Phosphorylation of MuSK S751D is significantly enhanced in response to sub-threshold levels of agrin. (3) Basal AChR phosphorylation is significantly increased as well as AChR phosphorylation after 15 minutes of agrin stimulation in MuSK S751D expressing muscle cells suggesting that the phosphomimetic mutant accelerates the downstream signalling. (4) Agrin-induced AChR clusters are bigger and disassemble more slowly in cells expressing MuSK S751D compared to cells expressing MuSK wild-type or MuSK S751A. (5) Myotubes expressing MuSK S751D form more microclusters than myotubes expressing MuSK wild-type or MuSK S751A.

Previous studies have shown that the activation loop carrying Y750, Y754, Y755 and the juxtamembrane region including Y553 are not only important for MuSK kinase activity but also responsible for autoinhibition. The crystal structure of unphosphorylated MuSK implied that the activation loop of MuSK provides a stringent autoinhibition to limit ligand-independent activation. In addition, a second autoinhibition mechanism involves the juxtamembrane region, which is largely disordered in MuSK and does not interact with the kinase domain^18^,^37^. This strong autoinhibition involving the juxtamembrane and the autoactivation loop is thought to be important for restricting MuSK activity to the synaptic site^18^,^38^. MuSK can overcome the stringent autoinhibition by binding to the cytoplasmic activator Dok7^19^. Thereby MuSK evolved a strategy that enables MuSK activation in the absence of agrin as required during prepatterning. Autoinhibition by the activation loop is a common mechanism among RTKs to control kinase activation^38^,^39^. In the inactive state the activation loop traverses the catalytic cleft, which blocks ATP binding. This closed conformation is stabilized by numerous hydrogen bonding interactions including bonding of Y754 to D724 and R728^18^. Studies on the insulin receptor have demonstrated that phosphorylation of the activation loop tyrosines Y1158, 1162 and Y1163 (equivalent to Y750, Y754 and Y755 in MuSK) results in destabilization of the autoinhibitory conformation and stabilization of the active conformation^40^. Full kinase activation represents a multi-step process with a specific order of autophosphorylation. Y754 in MuSK and Y1162 in the insulin receptor are the first tyrosines in the respective activation loop to become phosphorylated^18^,^40^. Wu and colleagues were able to provide a snapshot of the first phosphorylation event in the insulin-like growth factor 1 receptor kinase and insulin receptor using an ATP-competitive small-molecule inhibitor^41^. The structure indicates that E1159 (S751 in MuSK) and R1164 (K756 in MuSK) in the insulin receptor form a salt bridge, which appears to position Y1162 (Y754 in MuSK) in the active site for trans-phosphorylation. Phosphorylation of tyrosines, serines and threonines positively affects hydrogen bonding to donors such as arginine or lysine. It therefore appears possible that phosphorylation of S751 supports Y754 positioning in the active site for trans-phosphorylation. Likewise, mutating serine to aspartate might cause a similar effect since the strength of hydrogen bonding of aspartate is similar to phospho-serine^42^. Sustaining Y754 phosphorylation is expected to relief autoinhibition and/or to stabilize kinase activity. The EGFR, unlike other RTKs, adopts an active activation loop conformation, which does not require phosphorylation for biological activity. From the crystal structure of the EGFR kinase domain it was inferred that glutamate residues in its activation loop, in particular E848, can act as analogues of phosphorylated tyrosines found in other RTKs such as the insulin receptor^37^,^43^. Mutating S751 to D751 might therefore create a similar active conformation for the unphosphorylated activation loop of MuSK resulting in higher basal kinase activity. In muscle cells, MuSK S751D would therefore be able to escape the autoinhibition via the activation loop, consequently increasing agrin-independent MuSK signalling.

Lowered autoinhibition or respectively a stabilized active conformation of the activation loop might play an important role during two developmental states of NMJ development, namely AChR prepatterning and NMJ maintenance. NMJs have to be stable and functional for the entire lifetime of an organism^44^. However, very little is known about the mechanisms that control NMJ maintenance. Previous studies have shown that MuSK is required for NMJ stabilization and maintenance in adult mice^45^,^46^. We also know that MuSK and agrin are downregulated during postnatal development and both proteins are restricted to the synaptic region^47^,^48^. Therefore, one can assume that MuSK activation and signalling must be very efficient to keep the NMJ intact and functional. We propose that phosphorylation of S751 is part of a feedback loop that stabilizes MuSK activation. As discussed above, we think that phosphorylation of S751 eases autoinhibition by priming the activation loop for trans-phosphorylation, consequently increasing basal kinase activity. As a result, MuSK signalling would be adequately efficient even in the presence of low levels of agrin. Consistent with this model, we detect an enhanced phosphorylation of MuSK S751D in response to non-saturating concentrations of agrin.

Previously, Cheusova *et al*.^49^ reported CK2-dependent serine phosphorylation (S680, S697) in the kinase insert (KI) of MuSK. These phosphosites were not present in our MS analysis^15^. Serine phosphorylation was functionally linked to AChR cluster stability and maintenance without effect on MuSK kinase activity. The authors of the study speculated that serine residues in KI might provide a docking site for certain intracellular proteins such as 14-3-3γ, which could regulate AChR clustering through the actin cytoskeleton^49^,^50^. We do not favour the hypothesis that phosphorylation of S751 creates a protein binding site since mutation of S751 would destroy the binding site. It is expected that in such a case MuSK S751A and MuSK S751D would present the same phenotype. We however find that MuSK S751A and MuSK S751D behave differently in terms of kinase activity and AChR clustering efficiency.

Taken together, we identified a novel serine phosphorylation site in the activation loop of MuSK, which functions as regulatory site during kinase activation. We propose that phosphorylation of S751 lowers the autoinhibition of the activation loop. An increased basal kinase activity might play an important role during prepatterning or NMJ maintenance.

## Materials and Methods

### Constructs

Single amino acid substitutions were generated by site-directed mutagenesis using PCR. S751 residue in the rat MuSK cytoplasmic domain was either replaced with alanine (S751A) or aspartate (S751D). Constructs carrying a C-terminal HA-Tag were ligated into retroviral vector pBabe/puro^28^,^51^. S751D and S751A mutations were introduced into a previously described CMV/MuSK construct carrying a N-terminal HA-Tag^52^. Expression plasmids CMV/MuSK-WT, kinase active CMV/MuSK-KA (LS746/747MT) and kinase-dead CMV/MuSK-KD (K608A) have previously been described^16^,^17^,^52^. MuSK carrying a N-terminal HA-Tag and a C-terminal Myc-Tag was subcloned into LXSG to generate LXSG/MuSK-WT^13^,^21^,^28^. S751D and S751A mutations were introduced by exchanging the cytoplasmic MuSK region carrying S751 using ApaI and BglII.

### Antibodies and Reagents

The following antibodies were purchased from commercial sources: anti-phosphotyrosine PY99 (Santa Cruz Biotechnology, Dallas, TX) and PY-100 (Cell Signaling Technology, Leiden, The Netherlands), anti-AChR α (BD Biosciences), anti-AChR β (Sigma-Aldrich), anti-HA (Sigma-Aldrich), anti-Myc 9E10 (Sigma-Aldrich), anti-GFP (Santa Cruz), anti-Actin (BD Transduction Laboratories, Lexington, KT, USA), anti-Tubulin (Sigma-Aldrich). Biotin- and Alexa 594-conjugated α-bungarotoxin (BGT), Alexa 488-conjugated secondary antibodies were obtained from Invitrogen (Carlsbad, CA, USA). Horseradish-peroxidase-coupled goat anti-mouse/rat/rabbit secondary antibodies were purchased from Jackson ImmunoResearch (Ben Harbor, ME, USA). IRDye 680RD goat anti-mouse, IRDye 800CW goat anti-rabbit secondary antibodies were purchased form LI-COR (Bad Homburg, Germany). Streptavidin and Protein A agarose beads were obtained from Novagen (Merck KGaA, Darmstadt, Germany) and Roche (Roche Diagnostics, Mannheim, Germany), respectively. The antibody MuSK-EC (#83033) was used for immunohistochemistry and was previously described^53^. Polyclonal antibodies against the MuSK extracellular domain (Ig1–2) and anti-pY553, anti-pY754/755 were described previously^13^,^16^,^54^. For immunoprecipitation antibodies to the C-terminal sequence of MuSK were used as described previously^13^,^19^. Soluble neural A4B8 and non-neural A0B0 agrin were prepared as previously described^13^. Non-saturating concentrations of agrin were determined by stimulation of C2C12 myotubes with different amounts of agrin. MuSK phosphorylation was quantified and the agrin concentration inducing half maximal phosphorylation was used for experiments (equal to 1/5 of the saturating agrin concentration).

### Quantitative phosphoproteomics

Phosphorylation at MuSK serine 751 was identified in a previously published quantitative phosphoproteomic study^15^. A detailed description of sample preparation and data analysis can be found there. In brief, C2C12 cells were induced with agrin for 15, 60 and 240 minutes and compared to untreated C2C12 cells. Digested peptides were labelled with iTRAQ reagent (untreated with iTRAQ114, 15 min = iTRAQ115, 60 min = iTRAQ116, 240 min = iTRAQ117) and mixed in a 1:1:1:1 ratio^55^. Phosphorylated peptides were enriched by IMAC and subsequent TiO_2_. Resulting peptide mixtures were fractionated using high resolution Strong Cation Exchange (SCX) Chromatography on a 25 cm × 1 cm column. Phosphorylated peptides were separated into 130 fractions whereas unphosphorylated peptides were split into 70 fractions. SCX fractions were separated on a 25 cm reverse-phase C18 column using a 60-minute gradient on-line coupled to an LTQ Velos Orbitrap ETD mass spectrometer via a nano-electrospray source. The mass spectrometer was operated in data-dependent mode acquiring a full scan in the Orbitrap at resolution 60.000 followed by MS/MS scans of the five most abundant ions in the LTQ and in the Higher-energy collisional dissociation (HCD) cell. Raw files were processed in Proteome Discoverer (version 1.4.0.282). Database searches were performed using Mascot (version 2.2) against a concatenated target-decoy database based on the mouse UniProt database (version 2012_11). Oxidation of methionine and phosphorylation of serine, threonine and tyrosine were set as dynamic modifications and methylthio-cysteine and iTRAQ at the N terminus and lysine were specified as fixed modifications. Trypsin was defined as the proteolytic enzyme allowing for up to two missed cleavages. A mass tolerance of 7 ppm was set as the precursor ion tolerance. The fragment ion tolerance values for HCD and Collision-induced dissociation (CID) spectra were set to 0.03 and 0.5 Da, respectively. Reporter ion intensities were extracted in Proteome Discoverer from the closest centroid mass within an integration tolerance of 5 mmu. PhosphoRS (version 3.0) was employed to determine the localization of phosphorylated residues^56^.

### Generation and Affinity Purification of Polyclonal S751 Antibody

A small MuSK peptide of 17 amino acids length (GLSRNIYS(PO_4_)ADYYKADGC) with the pS751 residue at position 8 was synthesized by Fmoc-solid-phase synthesis, purified by high-pressure liquid chromatography and conjugated to a subunit of the carrier Keyhole Limpet Hemocyanin. 250 μg of antigen were used for the immunization of two rabbits. Reactivity of sera was tested by immunoblotting. The serum was purified by liquid chromatography. The column coated with the epitope peptide attached to maleimide activated POROS was washed with HEPES buffered solution (HBS), 12 mM HCl and afterwards re-equilibrated with HBS. Before injection to column, the serum was extracted with 1,1,2-Trichloro-1,2,2-trifluoroethane (Sigma-Aldrich) and filtrated with a 0.22 μm filter (Merck Millipore, CA, USA). After injection of serum, the column was washed with HBS until the baseline was stable. For the first elution step 1.5 M MgCl_2_, 50 mM sodium acetate pH 5.2 and for the second elution step 0.1 M glycine pH 2.45, 0.1 M NaCl was used. The eluates were buffered immediately with 2 M HEPES pH 7.9. Subsequently, the antibody solutions were dialyzed with phosphate buffered saline (PBS). The specificity of the antibodies was tested by immunoblotting (Fig. 4D) and immunohistochemistry (Supplementary Fig. S2).

### Cell Culture

HEK 293T cells were purchased from (ATCC, Manassas, VA, USA). Phoenix cells were kindly provided by Dr. Wilfried Ellmeier (Center for Physiology and Pathophysiology, Institute of Immunology, Medical University of Vienna, Austria). Cells were maintained in Dulbecco’s Modified Eagle’s Medium (DMEM) supplemented with glutamine, 4.5 mg/ml glucose, 10% fetal bovine serum (FBS) and 1% (v/v) penicillin/streptomycin at 37 °C and 5% CO2. HEK 293T cells were transfected using TurboFect (Thermo Fischer Scientific). Briefly, HEK 293T cells were plated on 6 well plates and transfected the next day with 2.2 μg DNA and 4 μl TurboFect in 200 μl DMEM. *MuSK*^−/−^ muscle cells were generated in the laboratory of Dr. Steven J. Burden (NYU School of Medicine, NYC, USA)^13^. Stable retroviral infection of *MuSK*^−/−^ muscle cells was performed using pBabe/MuSK-WT, S751D and S751A constructs. Cells were maintained and differentiated as described^13^,^16^. C2C12 myoblasts were originally obtained from the laboratory of Dr. Steven J. Burden (NYU School of Medicine, NYC, USA). Cells were grown and differentiated as described previously^57^. C2C12 muscle cells carrying the LXSG/MuSK constructs were generated by retroviral infection as previously described^13^. Briefly, Phoenix cells were transfected with plasmids using TurboFect. Virus-containing medium was collected two days after transfection and used immediately for the infection of C2C12 myoblasts in the presence of 2 μg/ml polybrene (Sigma-Aldrich). After 3 h the virus-containing medium was replaced with fresh growth medium. 24 h post-infection, muscle cells were split and maintained in growth medium. After passaging 2 times, myoblasts were sorted for GFP expression by fluorescence activated cell sorting followed by cell expansion for experiments. Muscle cells expressing MuSK/KA-2, MuSK/KD, MuSK Y553F and MuSK Y750, 754,755F were maintained as described previously^13^,^16^.

### Immunohistochemistry

Frozen muscle sections were thawed at room temperature, briefly rehydrated with PBS and blocked with 10% FBS in PBS for 30 minutes. Sections were incubated overnight at 4 °C with primary antibodies, washed three times with PBS and incubated at room temperature for 2 h with Alexa 488-conjugated secondary antibodies (Jackson Immunoresearch) and Alexa 594-conjugated α-BGT to label synaptic AChRs. Finally, the muscle sections were washed three times with PBS, post-fixed in 1% PFA for 5 min and mounted with Mowiol 4–88 (Sigma-Aldrich). The stained sections were imaged with Leica TCS SP5 confocal laser scanning microscope using 63x1.40 glycerol immersion magnification objective.

### Immunoprecipitation, AChR Pulldown, and Immunoblotting

Differentiated myotubes were starved for 2 h in DMEM and then stimulated with neural A4B8 agrin for 15, 60, 240, 480, 960 minutes. Cells were lysed in RIPA buffer (1% Triton X-100, 0.1% SDS, 0.5% sodium deoxycholate, 20 mM HEPES, pH 7.4, 150 mM NaCl, 2.5 mM EDTA) supplemented with protease (1 μg/ml leupeptin, 1 μg/ml pepstatin, 1 μg/ml aprotinin, and 0.2 mM PMSF) and phosphatase inhibitors (1 mM sodium orthovanadate, 50 mM sodium fluoride and 1 mM β-glycerophosphate). Cleared lysates were incubated with anti-MuSK (C-terminus) antibodies for MuSK isolation or with biotin-conjugated α-BGT for AChR pull down as previously described^16^. Following SDS-PAGE, proteins were transferred to PVDF membrane (Millipore, Darmstadt, Germany). The membrane was incubated with primary antibodies at 4 °C overnight. After incubation with secondary antibodies, signals were detected via chemiluminescence (Bio-Rad) on a ChemiDoc XRS + System (Bio-Rad, Hercules,CA).

### Surface Expression and Stability of MuSK in Muscle Cells

After 2 h of starvation, myotubes were stimulated with neural A4B8 agrin for 30 min. Cells were washed two times with PBS and then incubated for 60 min in a cold chamber (10–12 °C) with anti-HA antibodies diluted in 1% BSA, 140 mM NaCl, 20 mM HEPES, 1 mM CaCl_2_, 1 mM MgCl_2_, 5 mM KCL pH 7.4. Cells were washed with PBS two times for 5 min and lysed in 0.5% NP-40, 50 mM Tris/HCl pH 7.5, 1 mM EDTA, 100 mM NaCl supplemented with protease and phosphatase inhibitors. Protein A agarose was added for 1–3 hours. To analyse the stability of MuSK, myotubes were incubated with 7.5 ng/ml cycloheximide for 1, 2, 4, 6 h. Cells were lysed in RIPA buffer and proteins were analysed by immunoblotting. IRDye 680RD goat anti-mouse, IRDye 800CW goat anti-rabbit secondary antibodies were used and proteins were imaged with Odyssey Imaging System (LI-COR, Bad Homburg, Germany).

### AChR Clustering and Stability

To induce AChR clustering, myotubes were stimulated overnight (16 h) with neural A4B8 and non-neural A4B0 agrin. Next day, samples were either processed for staining or washed with PBS for three times and incubated with differentiation medium lacking agrin for 6 h and 12 h. To label surface AChRs, cells were fixed with 4% PFA in PBS at room temperature, washed two times with PBS for 5 min and incubated with Alexa 594-conjugated α-BGT in 2% FBS/PBS for 30 min. Cells were washed two times with PBS for 5 min and mounted with Mowiol 4–88. AChR clusters were imaged with a LEICA DM-IRB fluorescence microscope using 63x oil immersion magnification objective. Metamorph (Molecular Devices, Sunnyvale, USA) and ImageJ (NIH) software were used to acquire and quantify images.

### Quantification and Statistical Analysis

To quantify MuSK and AChR β phosphorylation, the ratio of phosphorylated protein to total protein was quantified using Image Lab Software (Bio-Rad, Hercules, CA). For the temporal activation profile, the values for MuSK-WT, S751D, S751A after 1 h agrin stimulation were defined as 1.0 and used to calculate the relative increase for the other time points. AChR clusters were quantified using ImageJ. In each image the myotube area was selected, thresholded and all objects with a minimum size of 4 μm^2^ were measured. Finally, mean of feret, number of clusters/1000 μm^2^ and myotubes with microclusters (as percentage of total myotubes) were statistically analysed using Graphpad Prism software (GraphPad Software Inc., CA, USA). AChR clustering experiments were replicated in at least three independent experiments (n = 3) and presented as the mean ± SEM. Data were analysed using non-parametric Kruskal Wallis, parametric one-way ANOVA for two or more group comparisons and two-way ANOVA for two factor comparisons with Dunn’s, Tukey’s post-tests and with Sidak’s multiple comparison tests, respectively. GraphPad Prism version 6.0 or R version 3.3.1 (RStudio, Boston, MA, USA) were used for analysis. The values of p < 0.05 (*), p < 0.01 (**), and p < 0.001 (***) were considered statistically significant.

## Additional Information

**How to cite this article**: Camurdanoglu, B. Z. *et al*. Musk Kinase Activity is Modulated By A Serine Phosphorylation Site in The Kinase Loop. *Sci. Rep.* **6**, 33583; doi: 10.1038/srep33583 (2016).

## Supplementary Material

Supplementary Information
