# Corrigendum: MuSK Kinase Activity is Modulated By A Serine Phosphorylation Site in The Kinase Loop

**DOI:** 10.1038/srep38271

**Published:** 2016-12-01

**Authors:** B. Z. Camurdanoglu, C. Hrovat, G. Dürnberger, M. Madalinski, K. Mechtler, R. Herbst

Scientific Reports 6: Article number: 3358310.1038/srep33583; published online: 09 26 2016; updated: 12 01 2016

The original version of this Article contained an error in the title of the paper, where the word “MuSK” was incorrectly given as “Musk”.

In addition, in the Supplementary Information file originally published with this Article, Figure S1 was omitted.

These errors have been corrected in the PDF and HTML versions of the Article, and the Supplementary Information file that now accompanies the Article.

