# Supplementary material for: Musk Kinase Activity is Modulated By A Serine Phosphorylation Site in The Kinase Loop
Source: Sci Rep. 2016 Sep 26;6:33583. doi: 10.1038/srep33583 (PMC5035991; doi:10.1038/srep33583)
Supplement: Supplementary Information [file srep33583-s1.pdf]

MUSK KINASE ACTIVITY IS MODULATED BY A SERINE PHOSPHORYLATION SITE IN THE KINASE LOOP

Camurdanoglu B.Z.[1], Hrovat C.[1], Dürnberger G.[3,4,5], Madalinski M.[3,4], Mechtler K.[3,4], Herbst R.[1,2,*]

[1] Center for Brain Research, Medical University of Vienna, Spitalgasse 4, 1090 Vienna, Austria.

[2] Institute of Immunology, Medical University of Vienna, Lazarettgasse 19, 1090 Vienna, Austria

[3] Institute for Molecular Pathology (IMP), Dr. Bohr-Gasse 7, 1030 Vienna, Austria

[4] Institute of Molecular Biotechnology (IMBA), Dr. Bohr-Gasse 3, 1030 Vienna, Austria

[5] Gregor Mendel Institute (GMI), Austrian Academy of Sciences, Vienna Biocenter (VBC), Dr. Bohr-Gasse 3, 1030 Vienna, Austria

SUPPLEMENTARY INFORMATION

SUPPLEMENTARY METHODS

Competition of anti-pS751 antibodies

Anti-pS751 antibodies were diluted in PBS / 10% FBS. Epitope peptides carrying pS751 (GLSRNIYS(PO_4)ADYYKADGC) or S751 (GLSRNIYSADYYKADGC) were added to the antibody solution at concentrations of 5 μM or 10 nM and incubated at room temperature for 30 min. The solutions containing antibodies and peptide were used for staining of muscle sections as described in the Methods section.

Co-immunoprecipitation of MuSK in Muscle Cells

Myotubes were starved for 2 h in DMEM and lysed in Co-IP Buffer (0.5% NP-40, 50 mM Tris/HCl pH 7.5, 1 mM EDTA, 100 mM NaCl) supplemented with protease and phosphatase inhibitors. Pre-cleared lysates were precipitated with anti-MuSK antibodies overnight. The next day protein A agarose was added for 1–3 h, the beads were washed three times for 10 min with Co-IP buffer. Precipitated lysates were analysed by immunoblotting using anti-Myc and anti-MuSK antibodies. For quantification, the ratio of Myc / MuSK signal was calculated.

Biotinylation assay

Differentiated myotubes were starved for 2 h in DMEM and stimulated with agrin (+, A4B8; −, A0B0) and subjected to biotinylation assay to quantify surface MuSK. Cells were washed two times with cold PBS supplemented with 0.1 mM $CaCl_2$, 1 mM $MgCl_2$ and then incubated for 20 min in a cold chamber (10-12°C) with freshly prepared 0.5 mg/ml EZ-Link Sulfo-NHS-LC-biotin (ThermoFisher Scientific) in PBS / 0.1 mM $CaCl_2$ /1 mM $MgCl_2$. Cells were washed with PBS / 0.1 mM $CaCl_2$ /1 mM $MgCl_2$ for three and quenched with cold DMEM for 10 min. Subsequently cells were rinsed with PBS and lysed in RIPA buffer (1% Triton X-100, 0.5% SDS, 0.5% sodium deoxycholate, 20 mM HEPES, pH 7.4, 150 mM NaCl, 2.5 mM EDTA) with 0.5% SDS supplemented with protease and phosphatase inhibitors. An aliquot was collected to detect total protein concentration, streptavidin agarose was added to the remaining samples and rotated for 3 hours at 4°C. Subsequently, samples were washed and proteins were subjected to SDS-PAGE. Samples were analysed by immunoblotting using anti-Myc antibodies and anti-insulin receptor (Becton Dickinson) antibody as a control. Total lysates were used as loading controls and immunoblotted with antibodies against Myc and Tubulin, respectively. For quantification, the ratio of surface MuSK to surface insulin receptor (Myc/IR) was calculated.

Figure S2

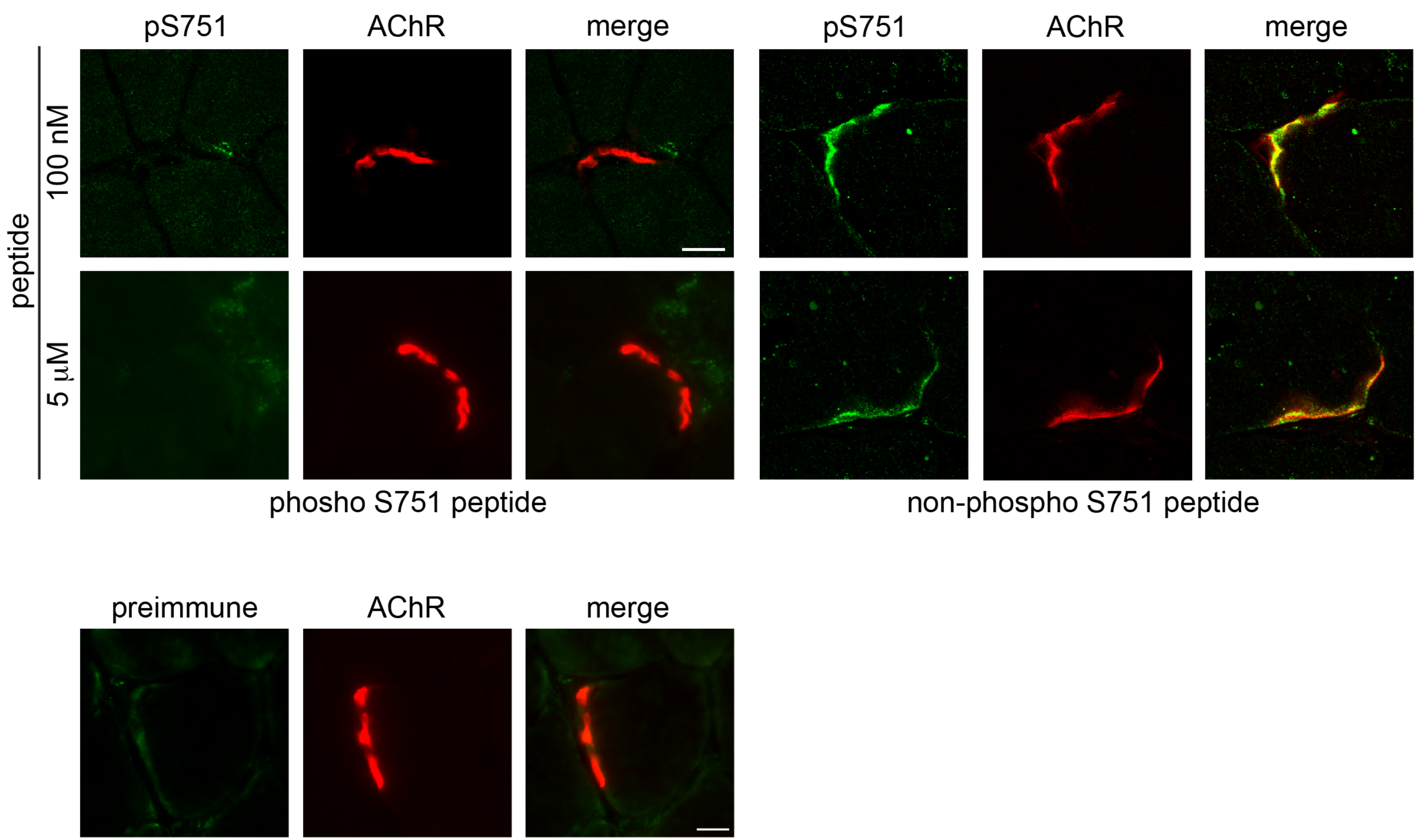

Supplementary Figure S2: Anti-pS751 antibodies specifically recognize MuSK pS751 in muscle tissue. Frozen sections of *M. gastrocnemius* were stained with anti-MuSK pS751 antibodies (green) that were pre-incubated incubated with different concentrations of antigenic peptide. Tissue was co-labeled with Alexa 594-conjugated α–BGT (red) to stain AChRs. Note that the staining of postsynaptic region with anti-MuSK pS751 antibodies is eliminated when pre-incubated with phospho S751 peptide but not when pre-incubated with non-phospho peptide. Additionally, pre-immune serum does not show any specific staining at NMJs. Images were obtained by confocal microscopy and representative images of are shown. Scale bar, 10 µm.

Figure S3

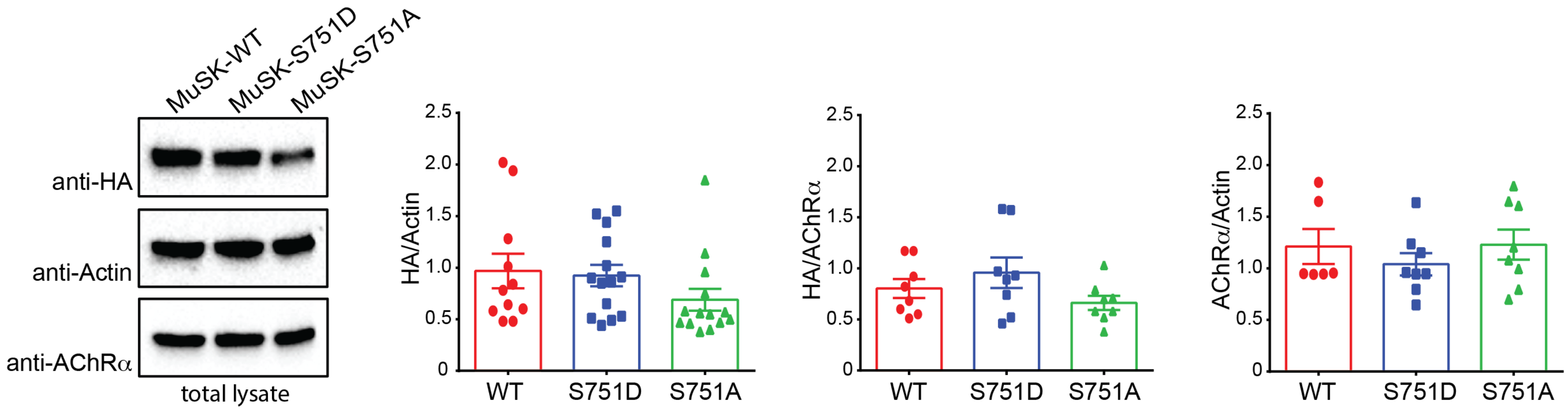

Supplementary Figure S3: Expression of wild-type and S751 mutant MuSK in *MuSK*$^{-/-}$ muscle cells. *MuSK*$^{-/-}$ muscle cells expressing MuSK wild-type and S751 mutant proteins were differentiated and subsequently subjected to cell lysis. Total lysates of mature myotubes were analyzed by immunoblotting using antibodies against anti-HA, anti-Actin and anti-AChR a, respectively. HA and AChR a signals were normalized to Actin. Note that cell lines express similar levels of MuSK and AChR a. Values are presented as the mean ± S.E.M. (Kruskal-Wallis with Dunn`s multiple comparison test, n = 9). WT, wild-type.

Figure S4

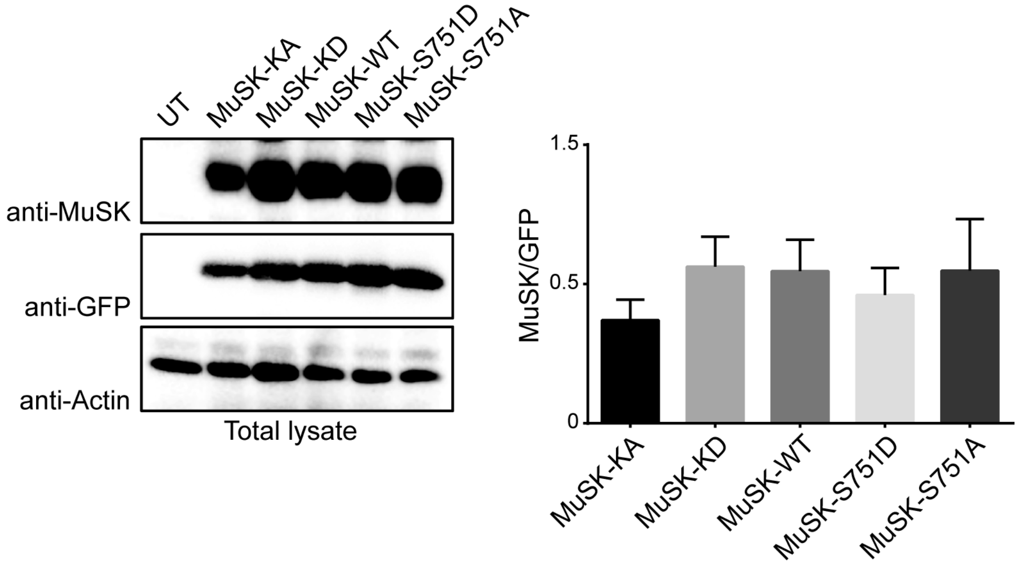

Supplementary Figure S4: Expression of wild-type and S751 mutant MuSK in heterologous cells. HEK 293T cells were transiently transfected with wild-type and mutant MuSK constructs together with GFP as an indicator of transfection efficiency. Two days after transfection, cell lysates were immunoblotted with anti-MuSK, anti-GFP and anti-Actin antibodies, respectively. MuSK signals were normalized to GFP. Note that cells express similar levels of different constructs. Values are presented as the mean ± S.E.M. (Kruskal-Wallis with Dunn`s multiple comparison test, n = 5). WT, wild-type; KA, kinase-active; KD, kinase-dead.

Figure S5

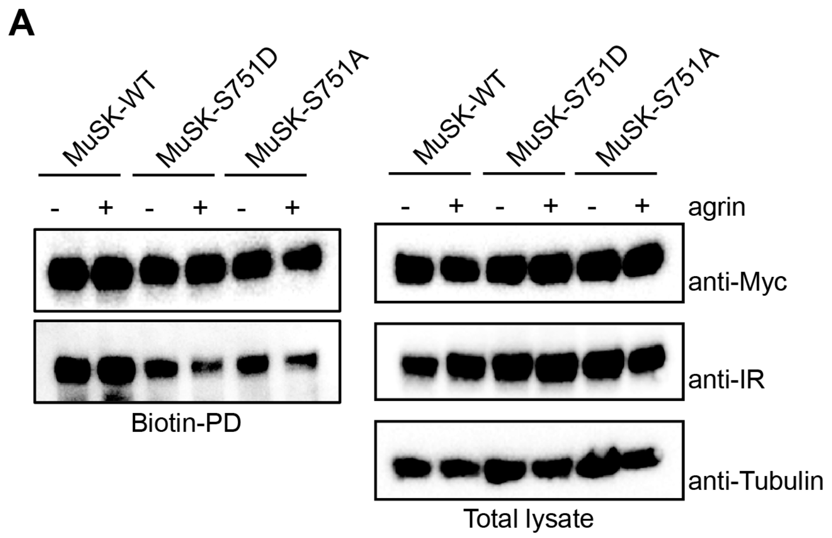

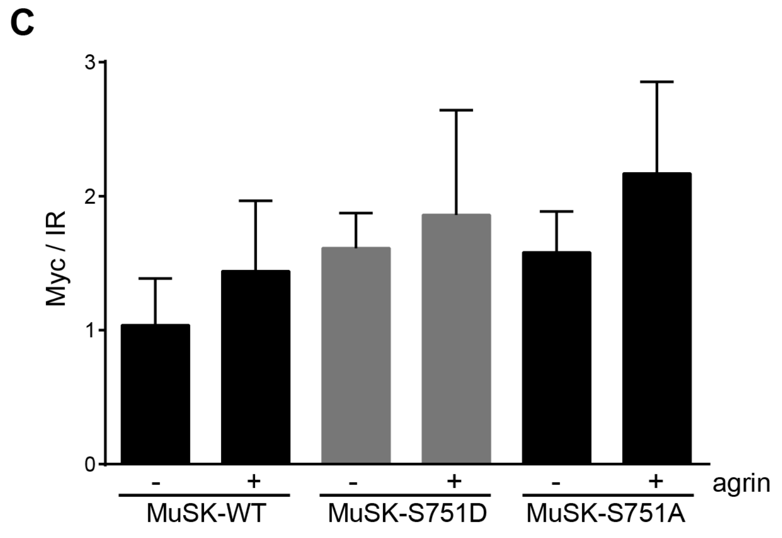

Supplementary Figure S5: MuSK surface is not affected by mutations in S751. (A) MuSK wild-type (WT), S751D, S751A constructs with a N-terminal HA-Tag and a C-terminal Myc-Tag were expressed in C2C12 muscle cells. Differentiated myotubes were stimulated with agrin for 30 min (+, A4B8; −, A0B0) and subjected to isolation of surface proteins via biotinylation followed cell lysis and by immunoblotting with anti-Myc and anti-insulin receptor (IR) antibodies, respectively. Total lysates were assayed by immunoblotting using antibodies against anti-Myc, anti-insulin receptor (IR) and anti-Tubulin, respectively. (B) Quantification of immunoblots is shown (Myc normalized to IR). Cell lines express similar levels of surface MuSK. Values are presented as the mean ± S.E.M. (One-way ANOVA with Tukey`s multiple comparison test, n = 4). PD, pull-down.

Figure S6

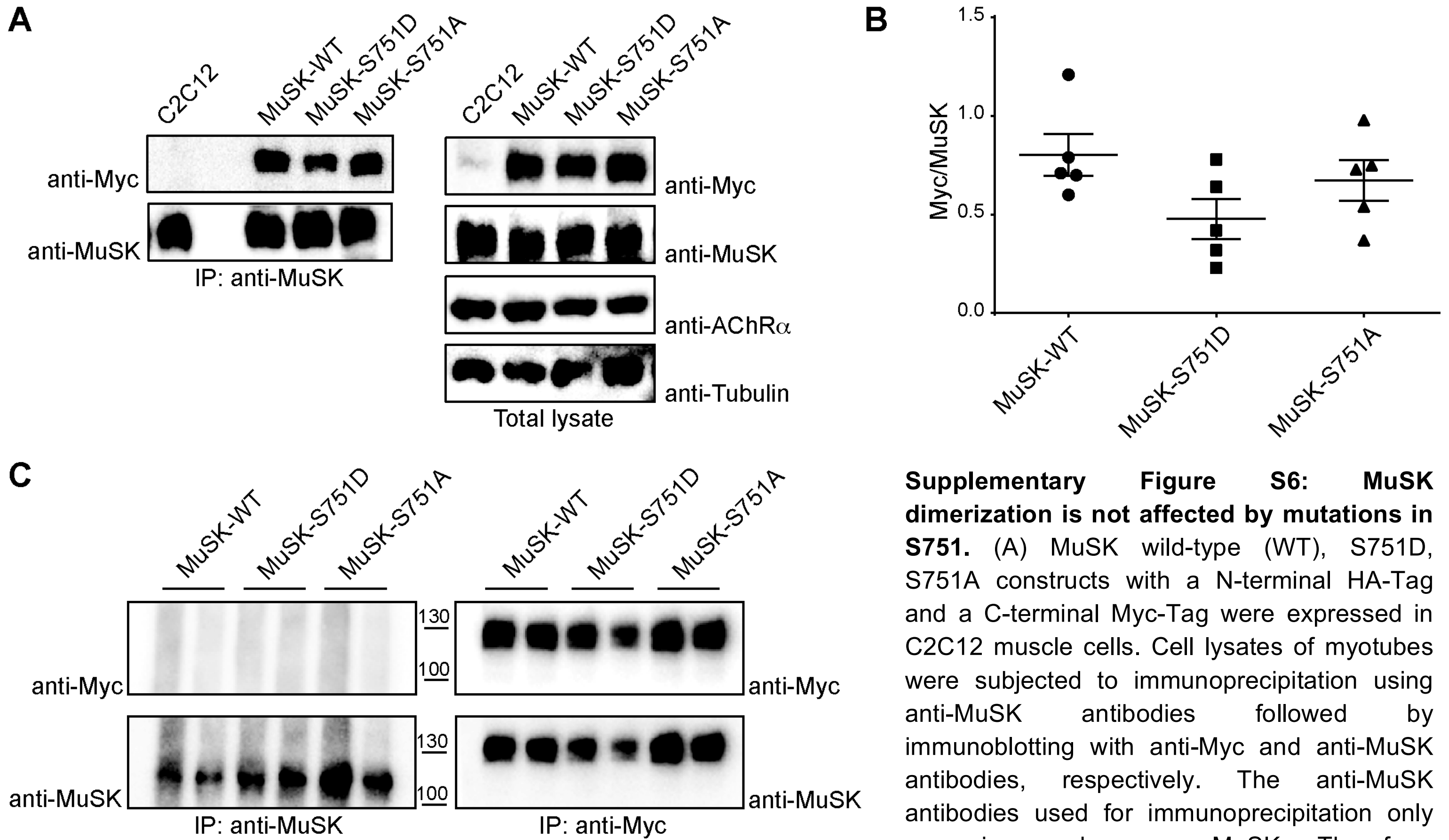

Supplementary Figure S6: MuSK dimerization is not affected by mutations in S751. (A) MuSK wild-type (WT), S751D, S751A constructs with a N-terminal HA-Tag and a C-terminal Myc-Tag were expressed in C2C12 muscle cells. Cell lysates of myotubes were subjected to immunoprecipitation using anti-MuSK antibodies followed by immunoblotting with anti-Myc and anti-MuSK antibodies, respectively. The anti-MuSK antibodies used for immunoprecipitation only recognize endogenous MuSK. Therefore, MuSK detected by immunoblotting with anti-Myc antibodies represents exogenous MuSK that is capable of interacting with endogenous MuSK. Total lysates were assayed by immunoblotting using antibodies against anti-Myc, anti-MuSK, anti-AChR a and anti-Tubulin, respectively. Note that there is no Myc signal in control C2C12 lysates (cells without expression of exogenous MuSK). (B) Quantification of immunoblots is shown (Myc normalized to MuSK). Similar amounts of exogenous wild-type and mutant MuSK were co-purified with endogenous MuSK. Values are presented as the mean ± S.E.M. (Kruskal-Wallis with Dunn`s multiple comparison test, n = 5) IP, immunoprecipitation. (C) Myotubes were lysed in RIPA buffer, which disrupts protein complexes. Cell lysates were subjected to immunoprecipitation using anti-MuSK or anti-Myc antibodies followed by immunoblotting with anti-Myc and anti-MuSK antibodies, respectively. Endogenous MuSK is purified with anti-MuSK antibodies only, whereas exogenous MuSK is recognized by anti-Myc antibodies only.

Figure S7

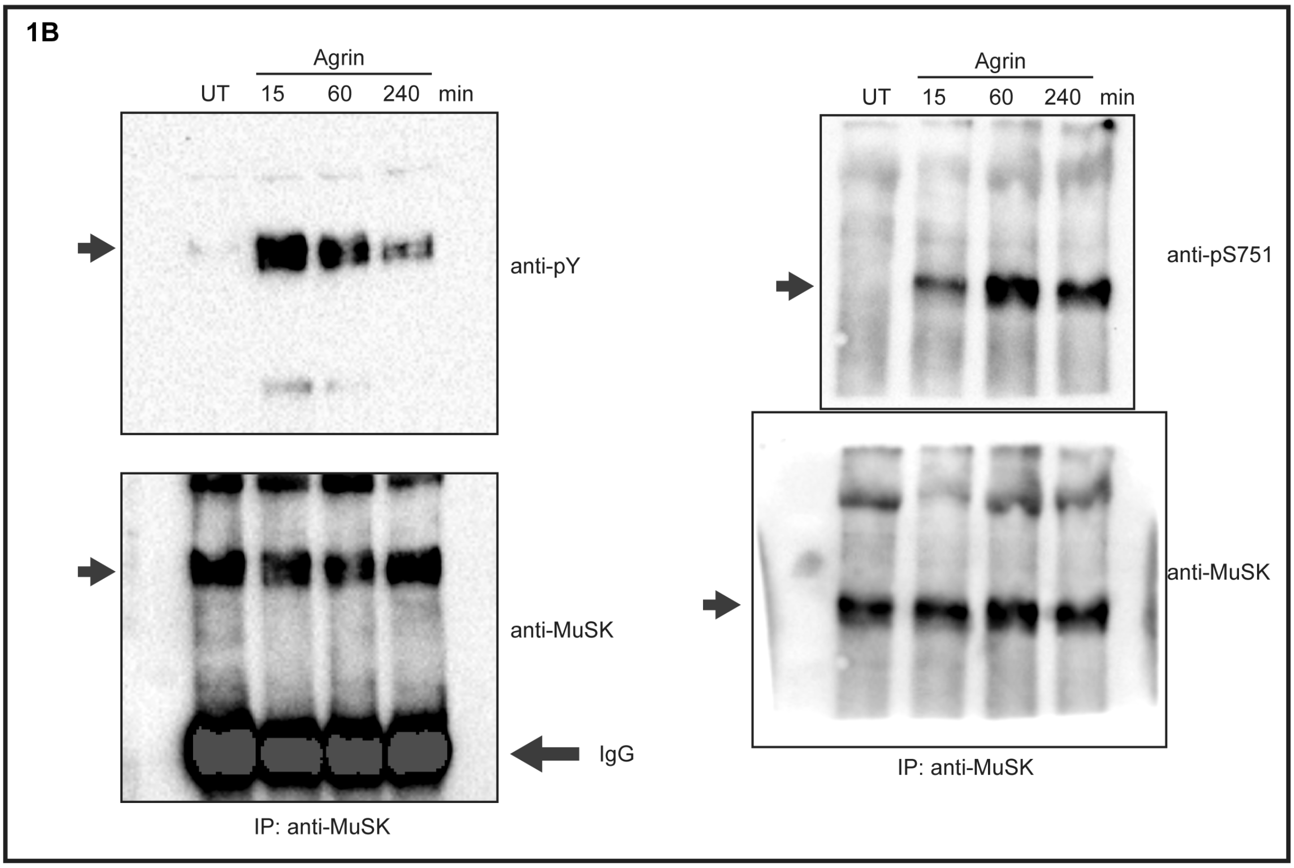

Supplementary Figure S7: S751 is phosphorylated in response to agrin. Original blots of the images shown in Figure 1. UT, untreated; IP, immunoprecipitation

Figure S8

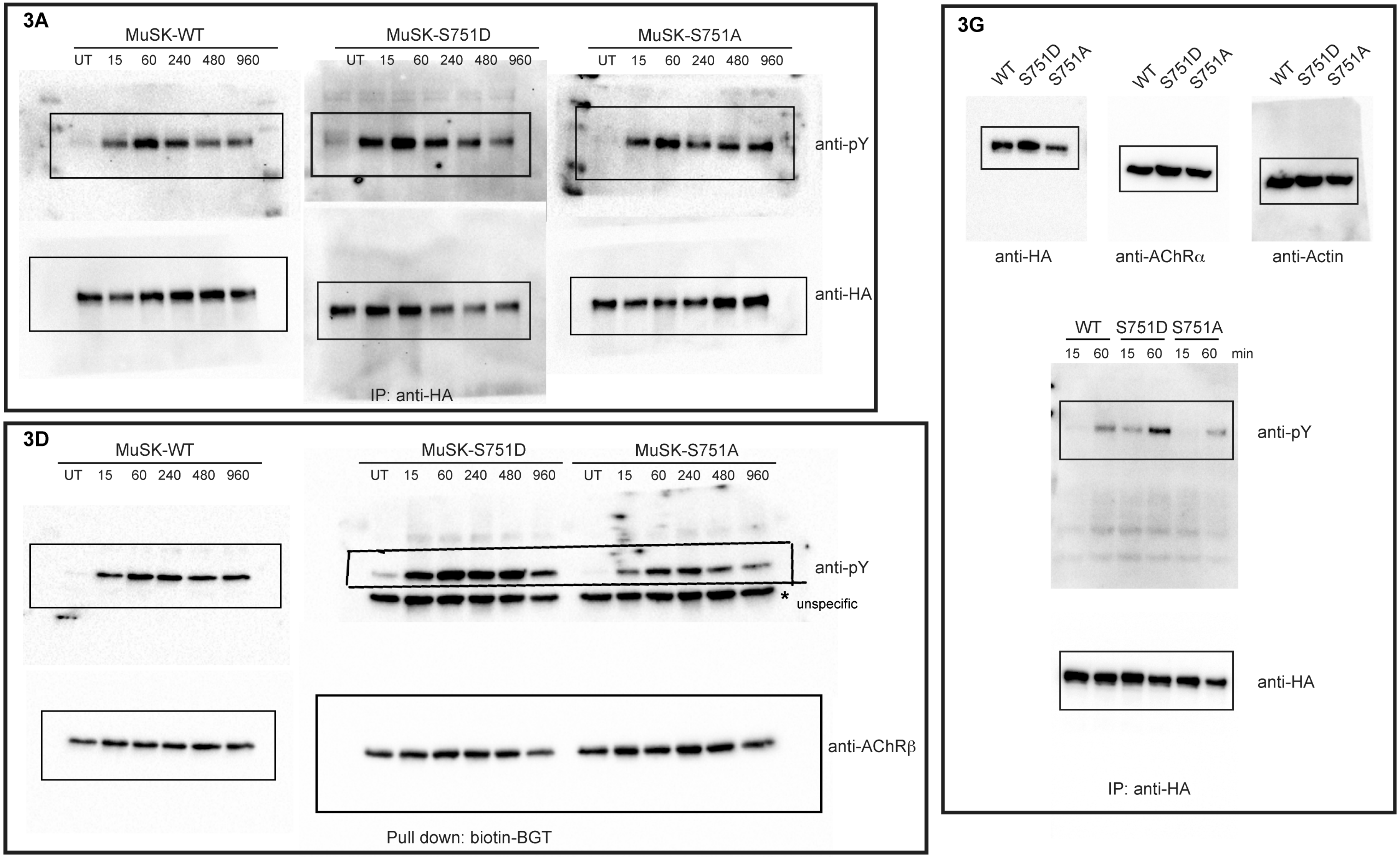

Supplementary Figure S8: A phosphomimetic S751 mutation modulates MuSK phosphorylation and causes increased AChR phosphorylation. Original blots of the images shown in Figure 3. UT, untreated; IP, immunoprecipitation

Figure S9

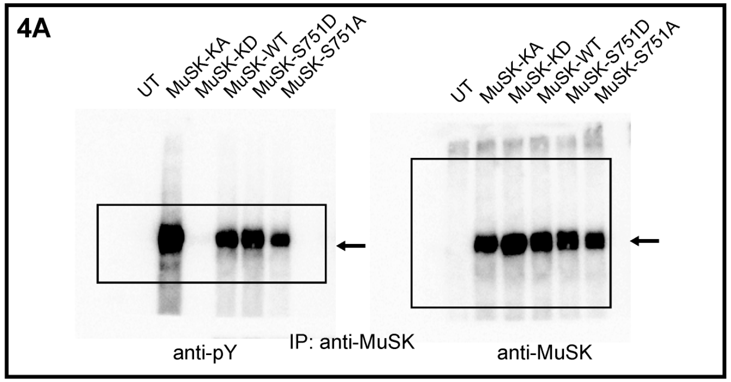

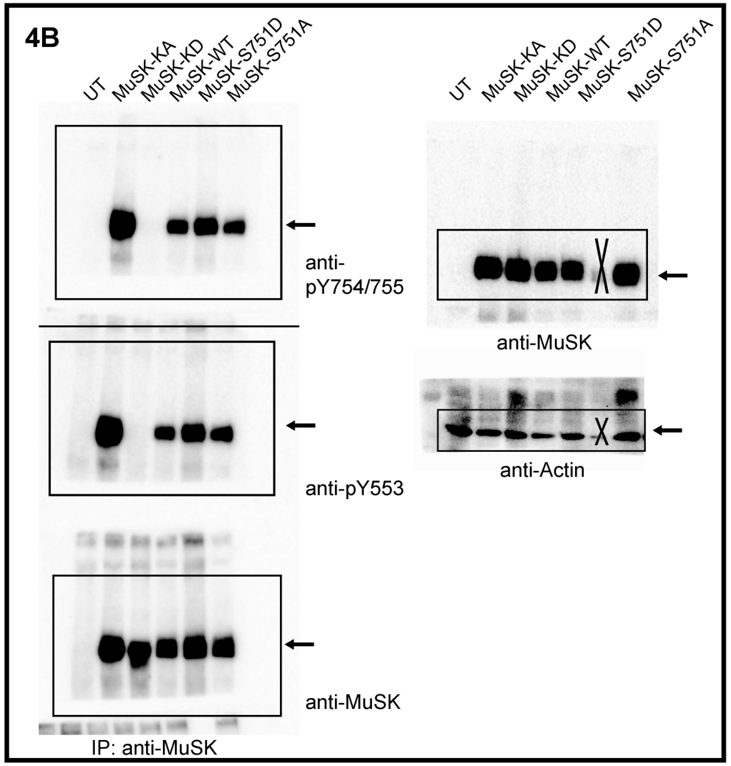

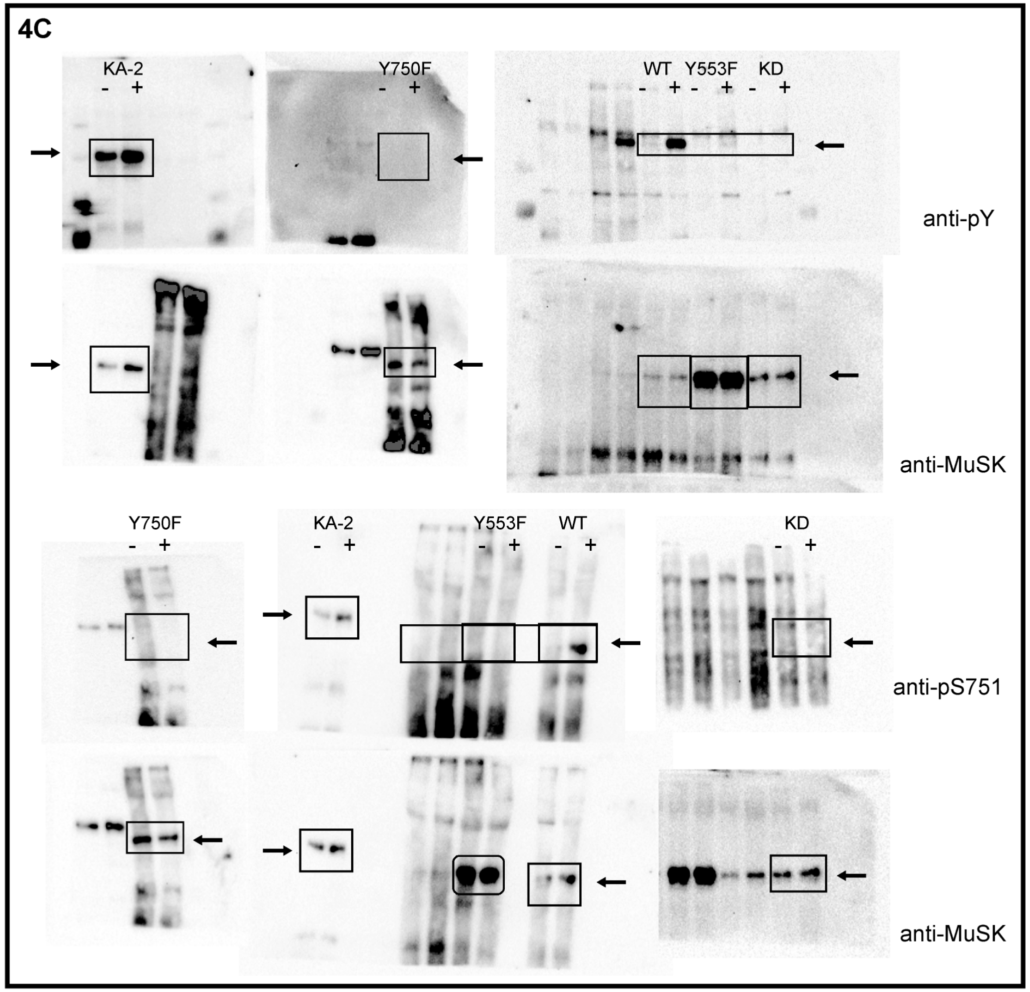

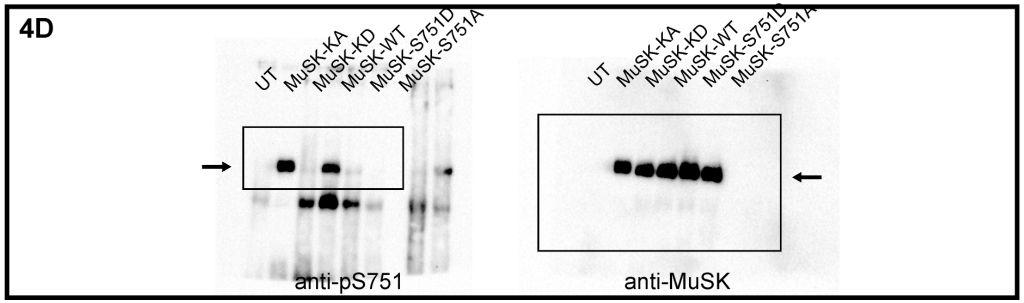

Supplementary Figure S9: S751 does not regulate autoactivation of MuSK in heterologous cells and phosphorylation of S751 is dependent on MuSK kinase activity. Original blots of the images shown in Figure 4. UT, untransfected; IP, immunoprecipitation

Figure S10

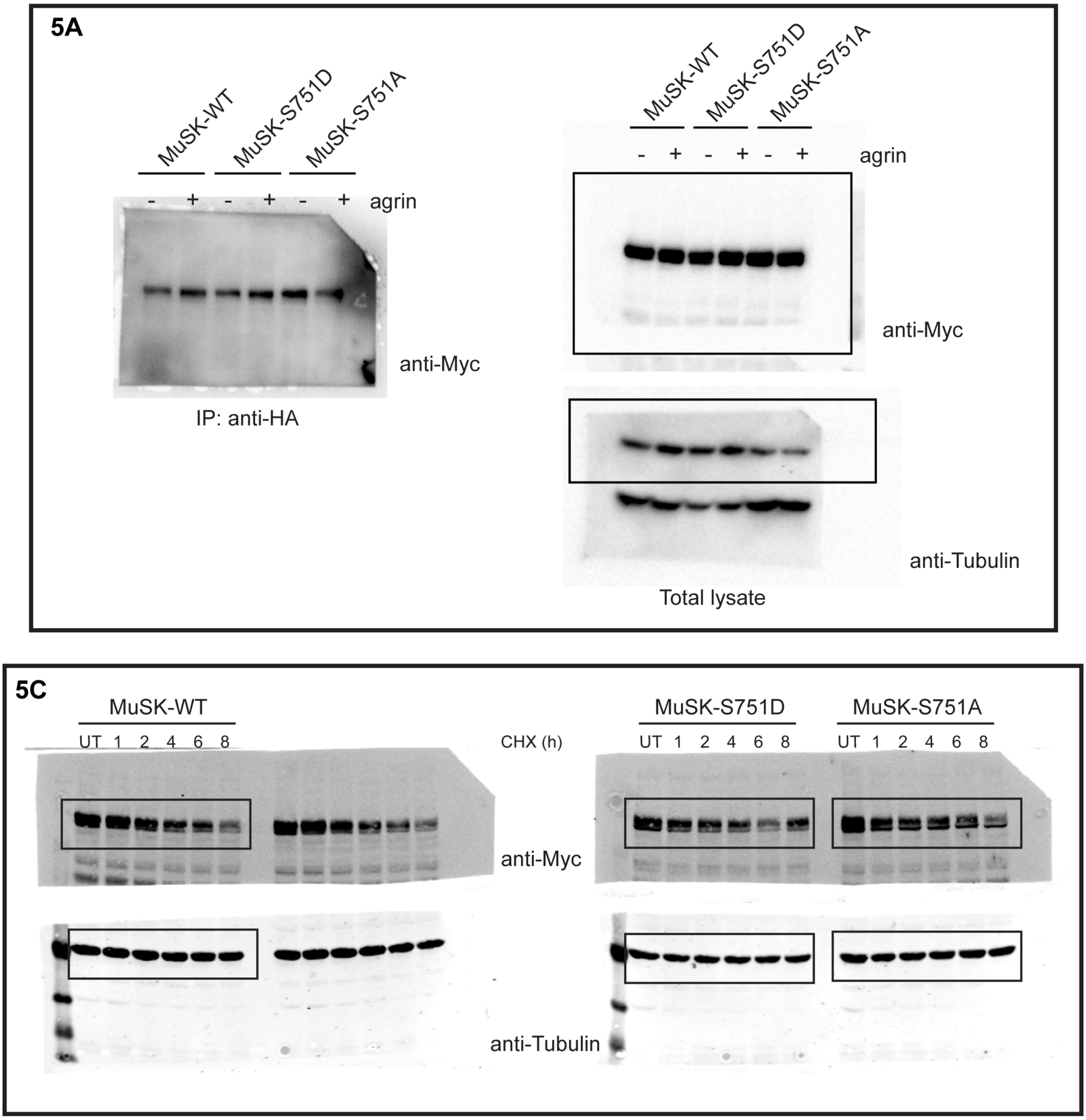

Supplementary Figure S10: Mutation of S751 does not alter MuSK surface expression and MuSK protein stability. Original blots of the images shown in Figure 5. UT, untreated; IP, immunoprecipitation
